# Neuronal hyperactivity–induced oxidant stress promotes in vivo α-synuclein brain spreading

**DOI:** 10.1126/sciadv.abn0356

**Published:** 2022-08-31

**Authors:** Michael Helwig, Ayse Ulusoy, Angela Rollar, Sinead A. O’Sullivan, Shirley S. L. Lee, Helia Aboutalebi, Rita Pinto-Costa, Benjamin Jevans, Michael Klinkenberg, Donato A. Di Monte

**Affiliations:** ^1^ German Center for Neurodegenerative Diseases (DZNE), Bonn 53127, Germany.; ^2^ Aligning Science Across Parkinson’s (ASAP) Collaborative Research Network, Chevy Chase, MD 20815, USA.

## Abstract

Interneuronal transfer and brain spreading of pathogenic proteins are features of neurodegenerative diseases. Pathophysiological conditions and mechanisms affecting this spreading remain poorly understood. This study investigated the relationship between neuronal activity and interneuronal transfer of α-synuclein, a Parkinson-associated protein, and elucidated mechanisms underlying this relationship. In a mouse model of α-synuclein brain spreading, hyperactivity augmented and hypoactivity attenuated protein transfer. Important features of neuronal hyperactivity reported here were an exacerbation of oxidative and nitrative reactions, pronounced accumulation of nitrated α-synuclein, and increased protein aggregation. Data also pointed to mitochondria as key targets and likely sources of reactive oxygen and nitrogen species within hyperactive neurons. Rescue experiments designed to counteract the increased burden of reactive oxygen species reversed hyperactivity-induced α-synuclein nitration, aggregation, and interneuronal transfer, providing first evidence of a causal link between these pathological effects of neuronal stimulation and indicating a mechanistic role of oxidant stress in hyperactivity-induced α-synuclein spreading.

## Teaser

Enhanced production of reactive oxygen species during neuronal hyperactivity exacerbates interneuronal α-synuclein transfer.

## INTRODUCTION

A prominent pathological feature of human neurodegenerative diseases is the intra- and/or extracellular deposition of proteinaceous aggregates (*1*). In Parkinson’s disease (PD), aggregates containing the protein α-synuclein (αS) are accumulated within neuronal cell bodies and neurites, forming typical inclusions known as Lewy bodies and Lewy neurites (*2*, *3*). Thorough assessment of Lewy pathology at early presymptomatic stages as well as during disease progression has yielded a number of intriguing observations. αS deposition not only affects neurons in the brain but also occurs within neurons of the peripheral nervous system (*4*–*7*). In the brain, specific anatomically interconnected regions are preferentially targeted by Lewy pathology, and the buildup of αS lesions often follows a stereotypical caudo-rostral pattern, advancing from the lower brainstem toward higher brain regions (*3*, *8*). Together, these pathological features prompted the hypothesis that cell-to-cell transfer of pathogenic αS species plays an important role in the progressive spread of αS lesions throughout the brain and between the brain and peripheral tissues (*9*–*12*). As a corollary to this hypothesis, much attention has been focused on neurons of the dorsal motor nucleus of the vagus (X^th^) nerve (DMnX) in the medulla oblongata (MO). These cholinergic cells are among the earliest sites of αS deposition during PD development and could therefore represent a source of initial pathological spreading (*3*, *8*). Furthermore, long efferent projections of DMnX neurons reach peripheral tissues through the vagus nerve, supporting a role of the DMnX as a relay center for peripheral-to-central (or central-to-peripheral) αS transmission (*13*–*15*).

Evidence of a relationship between interneuronal protein transfer and progressive spreading of pathological lesions underscores the relevance of investigations into pathophysiological conditions that may prompt or affect these processes. Growing experimental data are consistent with the ability of neuronal activity to modulate both the pathogenicity and interneuronal mobility of disease-associated proteins, namely, β-amyloid, tau, and αS. Chronic optogenetic neuronal stimulation and chemogenetic reduction of neuronal activity have been found to exacerbate and attenuate, respectively, β-amyloid peptide deposition (*16*–*18*). Similarly, in transgenic mouse models of human tau overexpression, tau pathology was enhanced and more widely spread under conditions of increased neuronal activity (*19*, *20*). In regard to αS, pharmacological induction of neuronal activity has been reported to promote αS aggregation and trafficking after “seeding” of organotypic brain slice cultures with αS preformed fibrils (PFFs) (*21*). Moreover, when PFFs were injected into the mouse dorsal striatum, motor deficits and αS pathology became more or less pronounced after chemogenetic induction of striatal hyper- or hypoactivity (*21*). The possibility that hyper- or hypoactivity may affect protein-induced pathology by modulating interneuronal protein transfer is further supported by data showing that secretion of β-amyloid, tau, or αS into the extracellular space and protein exchange from donor into recipient cells are clearly influenced by neuronal activity (*16*, *19*, *20*, *22*, *23*).

Despite increasing recognition of this pathophysiological role of neuronal activity, key questions concerning the relationship between hyper-/hypoactivity and protein spreading remain unanswered. First, very little is known about mechanisms by which changes in neuronal activity could modulate interneuronal protein transfer. Second, hyper- and hypoactivity not only affect protein spreading but also appear to influence the severity of aggregate pathology, raising the question of whether these activity-dependent changes are mediated through similar or different mechanisms (*16*, *19*, *21*). Last, it is conceivable that mechanisms underlying the relationship between neuronal activity and protein spreading may have a more or less pronounced impact on different neuronal populations, thus contributing to their discrete vulnerability to protein spreading and ensuing pathology. The aim of the current study was to further our understanding of these important mechanistic issues. In particular, experiments were designed to test the hypothesis that oxidant stress is a mechanism by which changes in neuronal activity can modulate interneuronal transfer and brain spreading of αS.

Several considerations prompted this hypothesis. A major cellular source of reactive oxygen species (ROS) is the mitochondrial electron transfer chain (ETC). During oxidative phosphorylation (OXPHOS), the flux of electrons through the ETC not only generates the energy gradient needed for adenosine triphosphate (ATP) production but also is accompanied by leakage of superoxide primarily from complex I and complex III (*24*–*26*). It is quite plausible therefore that, as a consequence of hyper- or hypoactivity, changes in neuronal energy demands could affect mitochondrial OXPHOS and electron transfer rates, resulting in increased or lowered ROS production. If hyper- or hypoactivity is associated with enhanced or reduced oxidant stress, the likelihood that this effect may ultimately impinge upon interneuronal protein spreading is supported by the results of an earlier investigation. These findings revealed that, under prooxidant conditions characterized by neuronal accumulation of both ROS and reactive nitrogen species (RNS), neuron-to-neuron transmission and consequent brain spreading of αS were significantly exacerbated (*27*). A potential relationship between neuronal activity, oxidant stress, and interneuronal αS transfer may be of particular relevance for DMnX neurons. These cells feature a distinct physiological trait that also characterizes other neuronal populations highly susceptible to αS pathology, including dopaminergic cells in the substantia nigra pars compacta and noradrenergic cells in the locus coeruleus (*28*–*31*). They autonomously generate a broad rhythmic action potential that is accompanied by slow oscillations of cytosolic calcium. This pacemaking activity creates a high basal metabolic demand, promotes calcium entry into mitochondria, and stimulates mitochondrial OXPHOS (*32*, *33*). It may also render these neurons particularly susceptible to activity-dependent oxidant stress and consequent protein spreading.

Experiments here were carried out using a unique paradigm of transgene expression targeting neurons in the DMnX-containing dorsal MO (DMnX-MO). This paradigm involved the use of transgenic Cre-inducible human αS (h-αS) knock-in mice and administration of adeno-associated viral vectors (AAVs) into the mouse vagus nerve. Intravagal treatment with AAVs carrying Cre recombinase DNA (Cre-AAVs) induced targeted h-αS expression that was followed by interneuronal transfer of the exogenous protein and its spreading from the dorsal MO toward higher brain regions. The intravagal route was also used for administration of AAVs delivering DREADD (Designer Receptor Exclusively Activated by Designer Drugs) DNAs. Targeted expression and pharmacologic stimulation of hyperactivity- or hypoactivity-inducing DREADDs allowed us to achieve tissue-specific changes in neuronal activity that markedly affected caudo-rostral h-αS spreading. Other findings revealed that increased protein spreading after neuronal hyperactivity was associated with pronounced ROS burden, nitrative modifications of both cytosolic and mitochondrial proteins, and enhanced h-αS assembly. Rescue experiments aimed at preventing neuronal ROS accumulation established a clear relationship between these pathological effects of neuronal stimulation and demonstrated a key mechanistic role of oxidant stress in hyperactivity-induced h-αS transfer.

## RESULTS

### Cre-induced expression of h-αS in the DMnX-MO of conditional transgenic mice triggered its caudo-rostral spreading

**Fig. 1.**
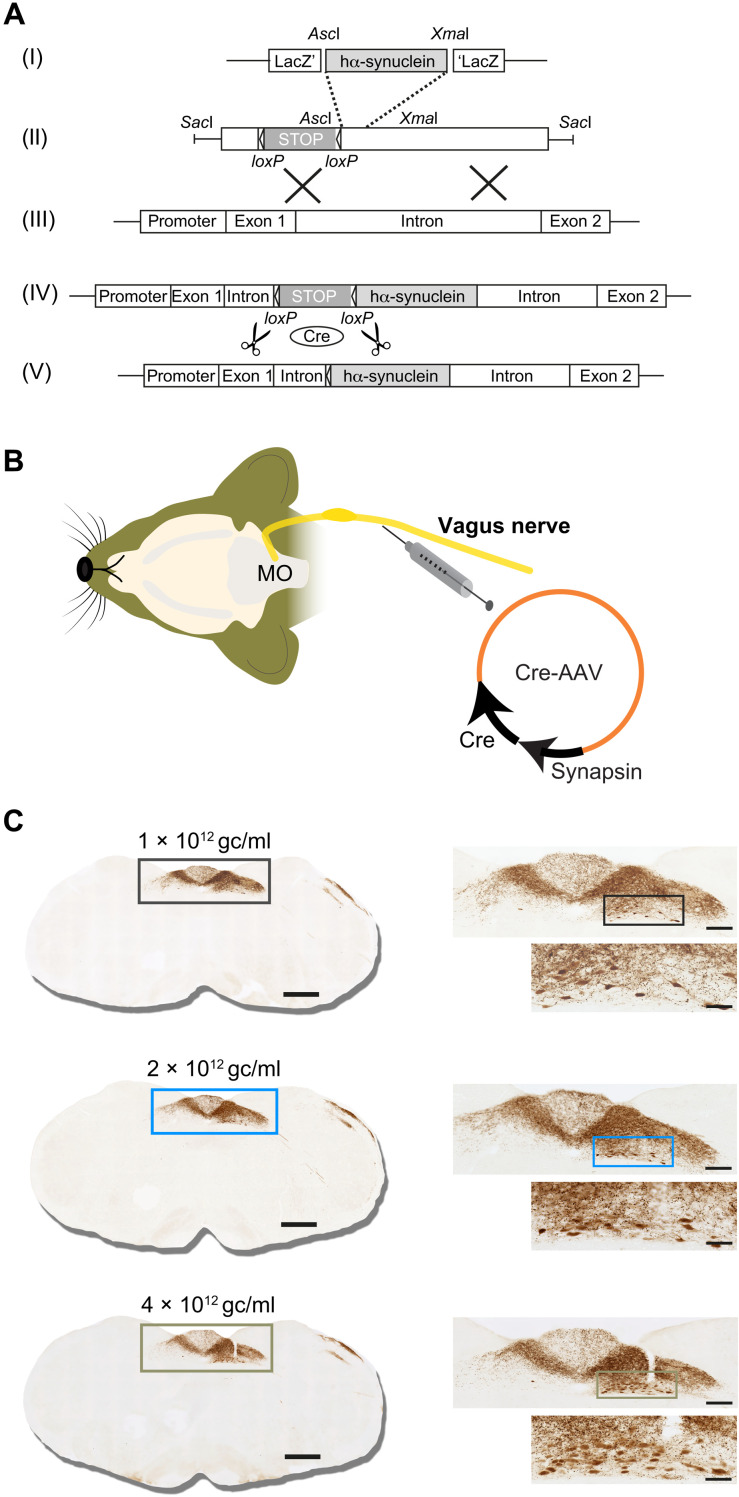
Cre-inducible expression of h-αS in the dorsal medulla oblongata of iR26-αS mice. (**A**) Gene targeting strategy for the generation of transgenic iR26-αS mice involved insertion of wild-type h-αS cDNA (I) into a Rosa26 targeting vector downstream to a *loxP*-flanked transcriptional termination (neo/STOP) cassette (II). Following homologous recombination at the murine *Rosa26* genomic locus (III), correctly targeted clones contained the neo/STOP cassette and the h-αS DNA sequence adjacent to the endogenous Rosa26 promoter (IV). In mice carrying this transgene, Cre recombinase–dependent excision of the neo/STOP cassette (V) drove the inducible expression of h-αS. (**B**) Transgenic expression of h-αS in the MO was induced by a unilateral injection of Cre-AAVs into the left vagus nerve. Gene expression was regulated by the human synapsin 1 promoter. (**C**) Mice were sacrificed at 4 weeks after a vagal injection of 1 × 10^12^ gc/ml (black boxes), 2 × 10^12^ gc/ml (light blue boxes), or 4 × 10^12^ gc/ml (green-brown boxes) of Cre-AAVs. Coronal sections of the MO were immunostained with anti–h-αS. Representative images show titer-dependent h-αS expression (brown staining) at low, medium, and high magnification. Boxes in the low-magnification images encompass an area of the dorsal MO that is shown at medium magnification. Boxes in the medium-magnification images encompass an area of the DMnX that is also shown at high magnification. Scale bars, 500, 200, and 50 μm in low-, medium-, and high-magnification images, respectively.

Targeted transgene insertion at the *Rosa26* locus on chromosome 6 is an effective strategy to generate transgenic mice and induce stable gene expression in these animals (*34*–*36*). Conditional expression can also be achieved by cloning the transgene into a Rosa26-targeting vector downstream to a *loxP*-flanked neo/STOP cassette (Fig. 1A, I to III). Under these conditions, gene expression would only occur upon Cre-mediated excision of the transcriptional termination sequence (Fig. 1A, IV and V). Transgenic Cre-inducible h-αS knock-in mice (iR26-αS) were generated using this approach, bred to homozygosity, and used for this study. To induce h-αS expression in the DMnX-MO, iR26-αS mice received a single unilateral injection of Cre-AAVs driving Cre recombinase expression under control of the human synapsin promoter into the left vagus nerve (Fig. 1B). At 4 weeks after this treatment, animals were sacrificed, their brains were dissected, and medullary tissue sections were stained with an antibody that specifically recognizes h-αS. Immunoreactivity showed a reproducible pattern of expression consistent with AAV-induced transduction of neuronal cell bodies in the DMnX and inferior vagal ganglion (*37*–*40*). H-αS–loaded perikarya and neurites were observed in the ipsilateral DMnX, while h-αS–positive axons originating from ganglionic cells projected into the nucleus of the tractus solitarius (NTS) both ipsi- and contralaterally (Fig. 1C). A dose-dependent effect of Cre-induced transgene expression was indicated by an increasing number of h-αS–positive DMnX neurons and increasing densities of immunoreactive NTS axons after injections with low, middle, and high AAV titers (Fig. 1C).

**Fig. 2.**
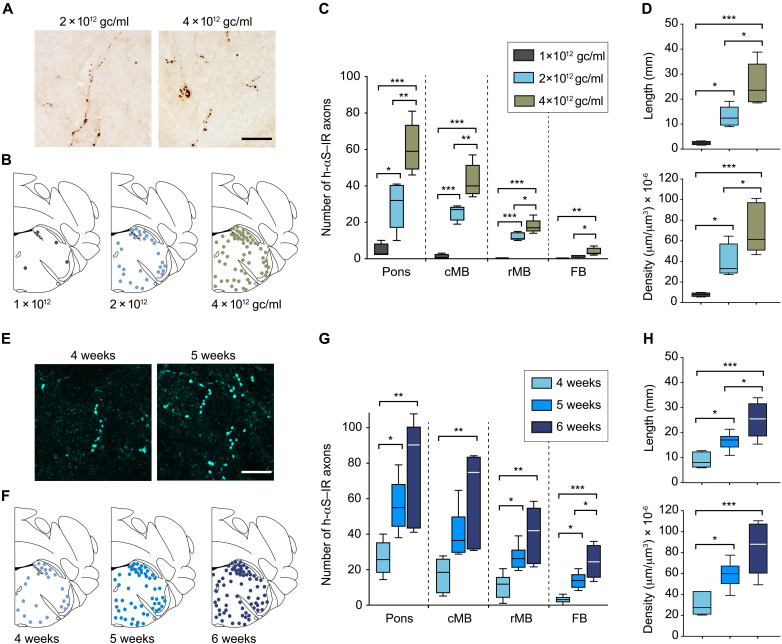
Caudo-rostral h-αS spreading is triggered by Cre-induced h-αS expression in the DMnX-MO. (**A** to **D**) iR26-αS mice received a single injection of 1 × 10^12^ gc/ml (*n* = 4, black), 2 × 10^12^ gc/ml (*n* = 5, light blue), or 4 × 10^12^ gc/ml (*n* = 5, green-brown) of Cre-AAVs into the left vagus nerve and were sacrificed 4 weeks later. Tissue sections were immunostained with anti–h-αS. Representative bright-field images show h-αS–positive axons in the left pons. Scale bar, 20 μm (A). Schematic plots of the distribution of h-αS–immunoreactive (h-αS–IR) axons in left pontine sections (bregma −5.40 mm) from three representative mice; each dot represents a separate single axon (B). The number of h-αS–immunoreactive axons was counted in tissue sections of the left pons, caudal midbrain (cMB), rostral midbrain (rMB), and forebrain (FB) (C). Length and density of h-αS–positive axons were estimated in a defined pontine area using the Space Balls stereological tool (D). (**E** to **H**) Mice received a single injection of Cre-AAVs (2 × 10^12^ gc/ml) into the left vagus nerve and were sacrificed 4 weeks (*n* = 6, light blue), 5 weeks (*n* = 6, blue), and 6 weeks (*n* = 5, dark blue) later. Representative confocal images show h-αS–positive axons (cyan) in tissue sections of the left pons. Scale bar, 20 μm (E). Schematic plots of the distribution of h-αS–immunoreactive axons in left pontine sections (F). The number of h-αS–immunoreactive axons was counted in tissue sections of the left pons, caudal midbrain, rostral midbrain, and forebrain (G). The length and density of h-αS–immunoreactive axons were estimated in a defined pontine area using the Space Balls stereological tool (H). Box and whisker plots show median, upper and lower quartiles, and maximum and minimum as whiskers. *P < 0.05, **P < 0.01, and ***P < 0.001.

To determine whether Cre-induced DMnX-MO expression of h-αS resulted in its interneuronal spreading toward more rostral brain regions, titer- and time-dependent experiments were carried out. First, iR26-αS mice received an intravagal injection of different titers of Cre-AAVs, i.e., 1 × 10^12^, 2 × 10^12^, or 4 × 10^12^ genome copies (gc)/ml, and were sacrificed at 4 weeks after administration. Coronal tissue sections were collected throughout the brain and stained with anti–h-αS. Spreading was assessed by counting the number of h-αS–immunoreactive axons in the pons, midbrain, and forebrain. Moreover, the length and density of h-αS–positive fibers were estimated in a defined pontine area encompassing the locus coeruleus and the nucleus parabrachialis using the Space Balls stereological probe. At 4 weeks after treatment, enlarged h-αS–positive axons with densely labeled and irregularly spaced varicosities could be detected in brain regions rostral to the MO (Fig. 2A). The number of these axons was highest in the pons and progressively lower in the caudal and rostral midbrain and forebrain, indicating that regions closer to the MO were more severely affected by the spreading pathology (Fig. 2, B and C). Axonal counts as well as Space Balls measurements were dependent upon the titer of Cre-AAV injections. Higher titers were associated with higher count, length, and density values, underscoring a relationship between the extent of AAV-induced h-αS expression and degree of protein transfer (Fig. 2, C and D).

For the time-course experiments, mice were injected with Cre-AAVs (2 × 10^12^ gc/ml) and sacrificed 4, 5, or 6 weeks after treatment. Results of these experiments provided further evidence of ascending protein spreading that became more and more pronounced at increasing time intervals. Axonal counts in the pons, midbrain, and forebrain and measurements of fiber length and density in pontine sections indicated a progressive buildup of h-αS–positive neurites between 4 and 6 weeks after AAV administration (Fig. 2, E to H). Primary sites of pathological h-αS accumulation included the coeruleus-subcoeruleus complex in the pons, the dorsal raphae and periacqueductal gray in the midbrain, the hypothalamus in the diencephalon, and the amygdala in the medial temporal lobe. Together, these results indicated that conditional expression of h-αS could be reproducibly achieved in the DMnX-MO of iR26-αS mice after a vagal Cre-AAV injection and that enhanced protein expression effectively triggered h-αS caudo-rostral brain advancement. On the basis of these initial findings, mice used in subsequent experiments were all injected with a Cre-AAV titer of 2 × 10^12^ gc/ml; h-αS spreading was then consistently assessed at 5 weeks after treatment.

### Increased activity of DMnX neurons exacerbated h-αS spreading

**Fig. 3.**
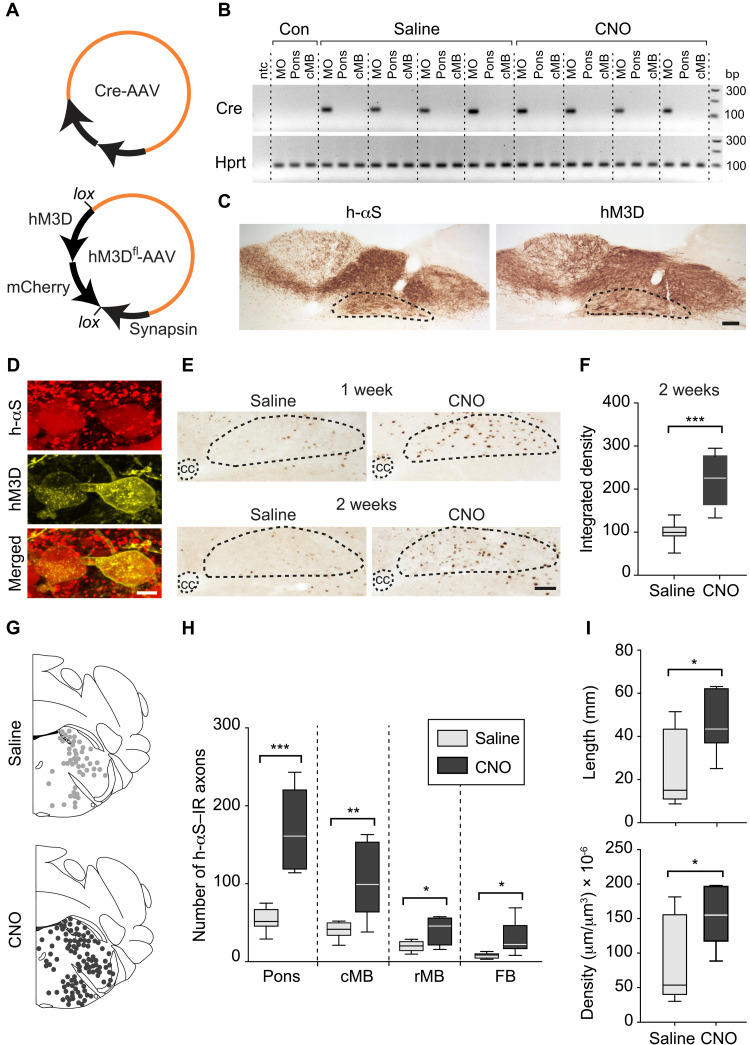
Neuronal hyperactivity exacerbates caudo-rostral h-αS spreading. (**A** and **B**) Mice were coinjected with Cre-AAVs together with AAVs designed for conditional hM3D DREADD expression (hM3D^fl^-AAVs) (A). They also received saline or CNO for 2 weeks (weeks 4 and 5) before sacrifice. Tissue specimens from the left dorsal MO, pons, and caudal midbrain were processed for RT-PCR; specific bands at 116 (Cre) and 90 (Hprt) bp; control samples (Con) from untreated mice; nontemplate control (ntc) (B). (**C** and **D**) Mice were coinjected with Cre- and hM3D^fl^-AAVs and treated with saline as above. Medullary sections were immunostained with anti–h-αS or anti-RFP; the DMnX is delineated. Scale bar, 100 μm (C). Confocal images of DMnX neurons double-labeled with anti–h-αS and anti-RFP. Scale bar, 10 μm (D). (**E** and **F**) Mice received an injection of Cre- and hM3D^fl^-AAVs and were treated with saline or CNO for 1 or 2 weeks. Medullary sections were labeled with anti–c-fos; the central canal (cc) and DMnX are delineated. Scale bar, 100 μm (E). Density measurements of c-fos immunoreactivity in the DMnX of mice injected with Cre- and hM3D^fl^-AAVs and treated with saline or CNO (*n* = 8 per group); data were calculated as percentage of the mean value in the saline group (F). (**G** to **I**) Mice received an injection of Cre- and hM3D^fl^-AAVs and were treated with saline (*n* = 6, gray) or CNO (*n* = 7, black). Brain sections were immunostained with anti–h-αS. Schematic plots of the distribution of h-αS–immunoreactive axons in left pontine sections (G). The number of h-αS–immunoreactive axons was counted in the left pons, caudal and rostral midbrain, and forebrain (H). The length and density of h-αS–immunoreactive axons were measured in pontine sections (I). Plots show median, upper and lower quartiles, and maximum and minimum as whiskers. *P < 0.05, **P < 0.01, and ***P < 0.001.

Expression of synthetically designed receptors and binding of these receptors with specific ligands can be used for transient activation or inactivation of targeted brain regions (*21*, *41*, *42*, *43*). This DREADD approach has been successfully applied to induce hyper- or hypoactivity of DMnX neurons that was validated using electrophysiological recordings and functional outcomes (*31*, *44*). Experiments were carried out here to assess the effects of chemogenetically induced DMnX hyperactivity on h-αS brain transfer. iR26-αS mice received an injection of a cocktail of two viral vectors into the left vagus nerve. The injected solution contained Cre-AAVs and AAVs designed to express Gq-coupled hM3D DREADD fused with mCherry under control of the human synapsin promoter; conditional expression was achieved using a double-floxed inverse ORF (DIO) (hM3D^fl^-AAVs; Fig. 3A). hM3D is a DREADD variant commonly used for neuronal excitation (*41*, *42*). Animals were kept for 5 weeks after AAV vagal administration and, starting at the beginning of week 4, also received a daily intraperitoneal injection of clozapine *N*-oxide (CNO), a synthetic ligand and DREADD activator. To define the distribution of AAV transduction and demonstrate confinement of AAVs within the MO, Cre recombinase mRNA was assessed by RT-PCR (reverse transcription polymerase chain reaction) as a marker of Cre-AAV–dependent transduction. Assays were carried out on tissue specimens of the dorsal MO, pons, and midbrain. RT-PCR products, when run on agarose gel, revealed clear bands for Cre recombinase mRNA in all samples from the DMnX-MO; quite in contrast, specimens from the pons and midbrain were consistently devoid of AAV-derived mRNA (Fig. 3B). No differences in Cre transduction were observed between saline- or CNO-treated mice (Fig. 3B). Expression of Cre recombinase was also assessed at the protein level by immunostaining medullary and pontine tissue sections from both saline and CNO-treated mice with a specific antibody against this protein. In all animals, reactivity with anti-Cre clearly labeled nuclei within neurons of the left (ipsilateral to the AAV injection side) DMnX; this observation sharply contrasted with findings in pontine sections, showing the absence of specific immunoreactivity (fig. S1).

Further analyses were performed using immunohistochemistry to verify that coadministration of Cre- and hM3D^fl^-AAVs resulted in Cre-induced expression of both h-αS and mCherry-fused hM3D proteins. For these analyses, coronal sections of the MO were stained with either anti–h-αS or an mCherry-recognizing antibody raised against red fluorescent protein (RFP). In all animals treated with Cre-/hM3D^fl^-AAVs plus saline or Cre-/hM3D^fl^-AAVs plus CNO, staining with either of the two antibodies showed robust DMnX-MO immunoreactivity, indicating successful expression of both h-αS and hM3D proteins (Fig. 3C shows images from a saline-injected mouse). Separate medullary sections were double-stained with anti–h-αS and anti-RFP and processed for immunofluorescence. In these sections, images of the DMnX showed signal colocalization within neuronal cell bodies that were immunoreactive for both h-αS and RFP (Fig. 3D). In contrast to findings in the MO, sections of the pons stained with anti-RFP were found to be consistently (e.g., in saline- or CNO-treated mice) devoid of immunoreactivity (fig. S2A). Furthermore, when pontine sections were double-labeled with anti–h-αS and anti-RFP, h-αS–loaded axons were observed, reflecting the MO-to-pons transfer of this protein; the same axons, however, showed no immunoreactivity for the RFP/mCherry protein (fig. S2B). Together, these observations further support the conclusion that AAV transduction and consequent protein expression targeted and remained strictly confined within medullary vagus–associated neurons.

Expression of the immediate early gene c-fos is an indirect marker of neuronal activity and was therefore used to assess DREADD activation upon treatment with its ligand and consequent neuronal hyperactivity. Samples analyzed for c-fos expression were obtained from mice injected with Cre-/hM3D^fl^-AAVs and then, after 3 weeks, treated with either saline or CNO for 1 or 2 weeks. Staining of medullary tissue sections with anti–c-fos revealed a significantly greater number of labeled DMnX neurons and significantly enhanced c-fos immunoreactivity as consequences of 1- or 2-week CNO administration, consistent with neuronal hyperactivity throughout the period of hM3D activation (Fig. 3, E and F). Next, treatment of iR26-αS mice with Cre-/hM3D^fl^-AAVs and subsequent injections with saline or CNO (during weeks 4 and 5 after AAV administration) were used to compare h-αS spreading in the absence or presence of hM3D activation. Tissue sections of the pons, caudal and rostral midbrain, and forebrain were stained with anti–h-αS and used for quantitative assessment of h-αS–immunoreactive axons. Axonal counts were consistently (i.e., in all brain regions) found to be much greater in CNO-treated as compared to saline-treated animals and, similarly, Space Balls measurements of pontine fiber length and density yielded values that were two to three times higher after hM3D activation (Fig. 3, G to I). Thus, hyperactivity had a pronounced effect on neuron-to-neuron protein transfer and significantly exacerbated h-αS advancement from the lower brainstem toward higher brain regions.

### Neuronal hyperactivity caused oxidant stress and accumulation of nitrated h-αS

**Fig. 4.**
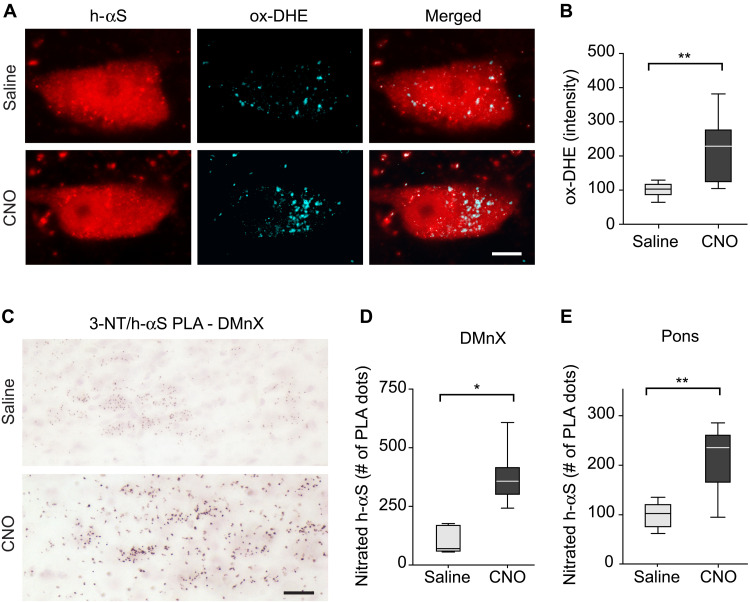
Neuronal hyperactivity induces oxidant and nitrative stress. (**A** and **B**) iR26-αS mice were coinjected with Cre- and hM3D^fl^-AAVs and treated with daily injections of either saline (*n* = 8) or CNO (*n* = 7) during weeks 4 and 5 after AAV injection. At the end of week 5, they received an injection of DHE 1 hour before sacrifice. Sections of the MO were immunostained with anti–h-αS. Representative confocal images show fluorescent puncta of ox-DHE within h-αS–positive DMnX neurons. Scale bar, 10 μm (A). Ox-DHE fluorescent intensity was measured within h-αS–positive DMnX neurons (approximately 30 neurons per animal). For each animal, neuronal intensity values were averaged; the averaged value was then calculated as percentage of the mean value in the saline group (B). (**C** to **E**) Mice injected with Cre- and hM3D^fl^-AAVs also received either saline (*n* = 6) or CNO (*n* = 7). To detect nitrated h-αS, sections of the MO and pons were processed for 3-NT/h-αS PLA. Representative images show specific chromogenic PLA dots in the left (injected side) DMnX. Scale bars, 50 μm (C). For each animal, the number of PLA dots was estimated in the left DMnX using unbiased stereology; data were calculated as percentage of the mean value in the saline group (D). The number of PLA dots was counted in left pontine sections; for each mouse, counts were done on a single section corresponding to bregma −5.34 mm, and data were calculated as percentage of the mean value in the saline group (E). Box and whisker plots show median, upper and lower quartiles, and maximum and minimum as whiskers. *P < 0.05 and **P < 0.01.

Experiments and analyses were carried out to determine whether increased neuronal activity was associated with oxidant stress, suggesting a relationship between hyperactivity, augmented ROS/RNS formation, and enhanced h-αS transfer. iR26-αS mice were injected with Cre-/hM3D^fl^-AAVs, treated for 2 weeks (weeks 4 and 5) with saline or CNO, and sacrificed at 5 weeks after AAV administration. One hour before the time of sacrifice, they also received an intraperitoneal injection of the superoxide indicator dihydroethidium (DHE). Microscopic examination of medullary sections from these mice revealed a punctate pattern of fluorescent signal within h-αS–loaded DMnX neurons (Fig. 4A). This signal is an indicator of the reaction between DHE and superoxide, leading to the formation of fluorescent ethidium cations (ox-DHEs) and their accumulation into mitochondria (*27*, *45*). Neurons displayed a wide range of number and intensity of fluorescent puncta, likely reflecting cell-to-cell variability of oxidant burden. Nevertheless, this punctate fluorescence was increased in sections from CNO-treated mice, and quantification of the intraneuronal ox-DHE signal revealed significantly higher intensity values after hM3D stimulation (Fig. 4, A and B).

During oxidant stress, enhanced ROS and RNS production may lead to nitration of αS at one or more of its four tyrosine residues (*46*). Accumulation of nitrated h-αS was therefore assessed in tissue sections of the MO of h-αS and hM3D coexpressing mice using a proximity ligation assay (PLA). For this assay, samples were incubated first with a pair of primary antibodies and then with secondary antibodies conjugated with PLA oligonucleotide probes; the two primary antibodies were anti–h-αS and anti–3-nitrotyrosine (3-NT), an antibody that recognizes 3-NT–modified protein residues (*27*). Following signal amplification, bright-field detection showed specific chromogenic dots in the left (ipsilateral to the vagal AAV injection) DMnX and revealed a marked signal enhancement in samples from CNO-treated as compared to saline-treated animals (Fig. 4C). When the number of PLA dots was estimated using unbiased stereological counting, this number was found to be increased by three to four times following stimulation of neuronal activity (Fig. 4D). 3-NT/h-αS PLA was then used to determine whether nitrated h-αS was also present in pontine tissue sections, i.e., within axons that accumulated h-αS as a result of MO-to-pons protein transfer. Specific labeling was scant in sections from saline-treated mice but more abundant after CNO administration, and stereological counts of pontine PLA dots were significantly higher in samples from the latter as compared to the former treatment group (Fig. 4E).

### Neuronal hyperactivity was associated with mitochondrial nitrative damage

**Fig. 5.**
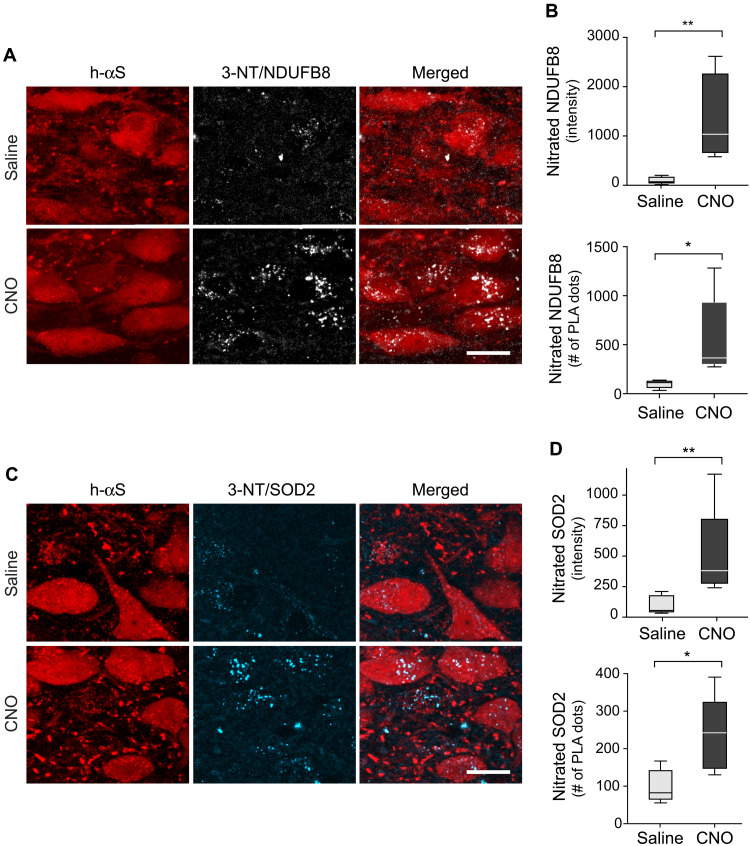
Mitochondrial nitrative damage within hyperactive h-αS–loaded neurons. (**A** to **D**) iR26-αS mice were injected intravagally with a solution containing Cre- and hM3D^fl^-AAVs, treated with either saline (*n* = 5) or CNO (*n* = 5) during weeks 4 and 5, and sacrificed at 5 weeks after AAV injection. To detect nitrated NDUFB8 (A and B) or nitrated SOD2 (C and D), sections of the MO were processed for either 3-NT/NDUFB8 or 3-NT/SOD2 PLA. They were also labeled with anti–h-αS. Representative confocal images show fluorescent PLA dots within h-αS–immunoreactive DMnX neurons. Scale bars, 20 μm (A and C). Fluorescent intensity and number of PLA dots were measured within h-αS–positive DMnX neurons. Approximately 15 neurons per animal were analyzed, and for each animal, intensity values and PLA counts were averaged. Data were then calculated as percentage of the mean value in the saline group (B and D). Box and whisker plots show median, upper and lower quartiles, and maximum and minimum as whiskers. *P < 0.05 and **P < 0.01.

To assess the involvement of mitochondria in hyperactivity-induced oxidative reactions, new PLA-based assays were developed and used for detection and quantification of nitrative modifications of key mitochondrial proteins. In particular, these analyses evaluated levels of nitrated mitochondrial complex I subunit NDUFB8 [NADH (reduced form of nicotinamide adenine dinucleotide) dehydrogenase (ubiquinone) I beta subcomplex subunit 8] and nitrated superoxide dismutase 2 (SOD2) within unstimulated versus hyperactive DMnX neurons. Medullary tissue sections from iR26-αS mice injected with Cre-/hM3D^fl^-AAVs and treated with saline or CNO were incubated with anti–3-NT and anti-NDUFB8 for assessment of nitrated NDUFB8 or with anti–3-NT and anti-SOD2 for the detection of nitrated SOD2. Samples were then incubated with oligonucleotide-labeled secondary antibodies and hybridizing connector oligonucleotides before amplification and visualization of the PLA signal with fluorescence detection. These sections were also stained with anti–h-αS. Confocal microscopy showed weak PLA signal in sections from saline-treated animals (Fig. 5, A and C), whereas robust labeling indicated accumulation of either nitrated NDUFB8 (Fig. 5A) or nitrated SOD2 (Fig. 5C) within h-αS–immunoreactive DMnX neurons after CNO administration. This marked effect of neuronal hyperactivity was confirmed by image analysis and quantification of intraneuronal PLA signal intensity as well as by counts of the PLA dots (Fig. 5, B and D). These findings not only provided additional evidence of oxidant stress within hyperactive neurons but also pointed to mitochondria as key targets of ROS/RNS-induced modifications during neuronal stimulation.

**Fig. 6.**
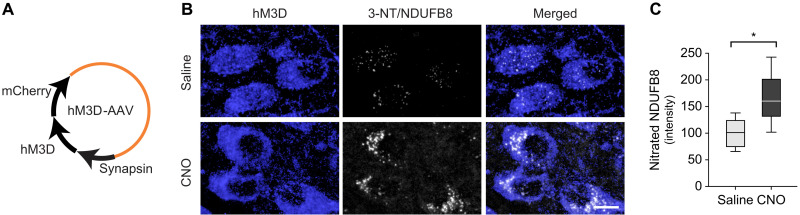
Nitration of mitochondrial NDUFB8 during neuronal hyperactivity in the absence of h-αS overexpression. (**A**) iR26-αS mice were injected with AAVs delivering (nonfloxed) hM3D fused with mCherry under control of the human synapsin promoter. (**B** and **C**) Mice received an intravagal injection of hM3D-AAVs and were then treated with daily injections of either saline (*n* = 5, gray bar) or CNO (*n* = 5, black bar) during weeks 4 and 5 after AAV injection. They were then sacrificed at the end of week 5. Medullary tissue sections were processed for fluorescent microscopy and double-labeled with 3-NT/NDUFB8 PLA and anti-RFP (for the detection of mCherry-tagged hM3D). Representative confocal images show fluorescent PLA dots within hM3D/RFP-positive DMnX neurons. Scale bar, 10 μm (B). Fluorescent intensity was measured within hM3D/RFP-positive DMnX neurons. Approximately 40 neurons per animal were analyzed, and for each animal, intensity values were averaged and calculated as percentage of the mean value in the saline group. Box and whisker plots show median, upper and lower quartiles, and maximum and minimum as whiskers. *P < 0.05 (C).

Evidence of oxidant stress after stimulation of h-αS–loaded DMnX neurons raised the question of whether ROS/RNS burden is a direct consequence of neuronal hyperactivity or represents a specific feature of neuronal stimulation in the presence of enhanced h-αS expression. To address this question, a separate set of experiments was designed using iR26-αS mice that received an intravagal injection of AAVs carrying nonfloxed hM3D DNA (Fig. 6A). This treatment aimed at expressing the excitatory DREADD in the absence of concurrent h-αS expression. At 3 weeks after AAV treatment, the animals were divided into two groups that received daily saline or CNO injections for 2 weeks before being sacrificed. Medullary tissue sections were processed for fluorescent microscopy and double-labeled with 3-NT/NDUFB8 PLA and anti-RFP. Microscopic examination revealed an overt increase in PLA signal in samples from CNO-treated mice (Fig. 6B). Similarly, when PLA signal intensity was measured within RFP-positive DMnX neurons and compared between saline- and CNO-injected animals, significantly higher fluorescence characterized the latter set of samples (Fig. 6C). Thus, neuronal hyperactivity was itself capable of inducing nitrative modifications of mitochondrial NDUFB8.

### Accumulation of nitrated h-αS and protein spreading were reduced after suppression of neuronal activity

**Fig. 7.**
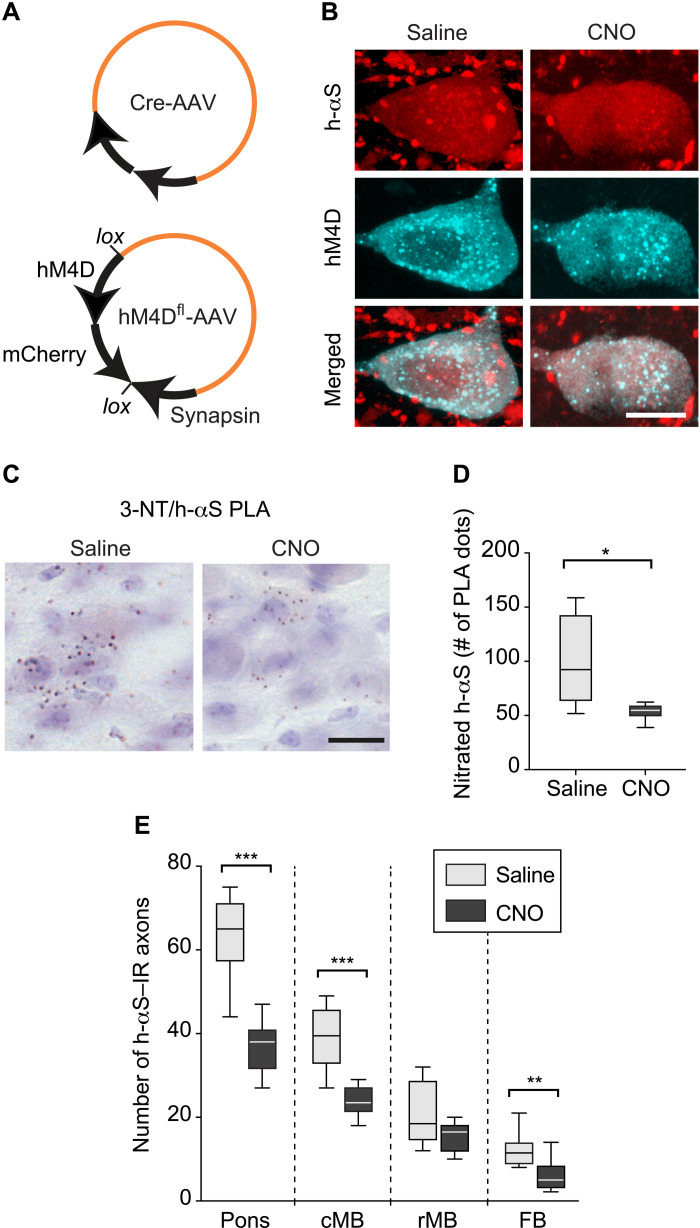
Caudo-rostral h-αS spreading is reduced after suppression of neuronal activity. (**A**) iR26-αS mice were injected with Cre-AAVs together with AAVs designed to express Gi-coupled hM4D DREADD fused with mCherry under control of the human synapsin promoter; conditional expression was achieved using a DIO system (hM4D^fl^-AAVs). (**B**) Mice received an injection of Cre- and hM4D^fl^-AAVs and were then treated with daily injections of either saline or CNO during weeks 4 and 5 after AAV treatment. They were then sacrificed at the end of week 5. Medullary sections were double-labeled with anti–h-αS and anti-RFP and processed for confocal microscopy. Scale bar, 10 μm. (**C** and **D**) Mice were injected with Cre- and hM3D^fl^-AAVs and received either saline (*n* = 6) or CNO (*n* = 6). Tissue sections of the MO were processed for 3-NT/h-αS PLA and counterstained with Nissl; representative images show specific chromogenic PLA dots in the left DMnX. Scale bar, 20 μm (C). For each mouse, the number of PLA dots was stereologically counted in the left DMnX; data were calculated as percentage of the mean value in the saline group (D). (**E**) Mice received an injection of Cre- and hM4D^fl^-AAVs and were then treated with either saline (*n* = 8) or CNO (*n* = 10). Tissue sections throughout the brain were immunostained with anti–h-αS, and the number of h-αS–immunoreactive axons was counted in predefined tissue sections of the left pons, caudal midbrain, rostral midbrain, and forebrain. Box and whisker plots show median, upper and lower quartiles, and maximum and minimum as whiskers. *P < 0.05, **P < 0.01, and ***P < 0.001.

Expression and CNO-induced activation of the DREADD hM4D result in membrane hyperpolarization and suppression of neuronal activity (*31*, *41*, *42*). Experiments were therefore carried out using hM4D expression as a tool for assessing the effects of neuronal hypoactivity on h-αS nitration and h-αS spreading. Mice received an intravagal coinjection of Cre-AAVs and AAVs designed to express Gi-coupled hM4D DREADD fused with mCherry under control of the human synapsin promoter; conditional expression was achieved using a DIO system (hM4D^fl^-AAVs, Fig. 7A). They were also treated for 2 weeks (weeks 4 and 5 after AAV injection) with saline or CNO before being sacrificed at 5 weeks after AAV administration. Postmortem analyses of medullary tissue sections that were double-labeled for h-αS and RFP showed colocalization of immunoreactivities within DMnX neurons, thus confirming cotransduction and coexpression (Fig. 7B). To evaluate potential changes in h-αS nitration, tissue sections of the MO were processed for 3-NT/h-αS PLA. Microscopic examination revealed that the number of PLA-specific chromogenic dots was reduced in the DMnX of CNO-treated as compared to saline-treated mice (Fig. 7C). Similarly, when the number of these PLA dots was estimated by unbiased stereological counting, a significant decrease in h-αS nitration was seen after CNO administration (Fig. 7D). Coronal tissue sections of the pons, caudal and rostral midbrain, and forebrain were then immunostained with anti–h-αS, and the presence of h-αS–positive axons was evaluated in these samples as evidence of caudo-rostral protein spreading. As shown in Fig. 7E, axonal counts yielded numbers that were consistently lower in all regions of the brain of CNO-injected animals, indicating that caudo-rostral protein spreading was attenuated as a result of neuronal hypoactivity targeted to the DMnX-MO. Thus, hypoactivity was associated with effects that were the opposite of those seen after induction of hyperactivity, further supporting a relationship between neuronal activity, ROS/RNS production, and h-αS spreading.

### Enhanced SOD2 expression reversed hyperactivity-induced h-αS spreading

**Fig. 8.**
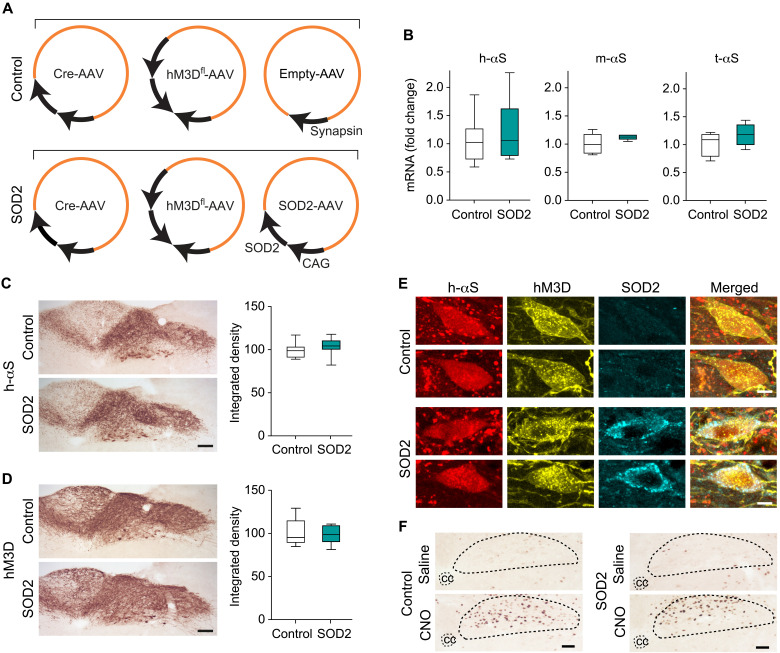
H-αS, hM3D, and SOD2 are coexpressed after AAV-induced transduction. (**A**) iR26-αS mice were coinjected with Cre- and hM3D^fl^-AAVs together with either empty AAVs or AAVs delivering human SOD2 under control of the CAG promoter. (**B**) Mice treated with Cre-, hM3D^fl^-, and empty-AAVs (control mice, *n* = 8) and animals treated with Cre-, hM3D^fl^-, and SOD2-AAVs (SOD2 mice, *n* = 6) were sacrificed at 5 weeks after AAV injection. Levels of h-αS, mouse αS (m-αS), total (human + mouse) αS (t-αS), and, as housekeeping control, Hprt mRNAs were measured in the left DMnX-MO by qPCR. (**C** and **D**) Coronal sections of the MO from control (*n* = 10) and SOD2 (*n* = 10) mice were immunostained with either anti–h-αS or anti-RFP (for detection of mCherry-tagged hM3D). Representative images show robust immunoreactivity of either protein in the DMnX-MO. Scale bars, 100 μm. Integrated density measurements were performed in the left (injected side) DMnX; data were calculated as percentage of the mean value in the control group. (**E**) Coronal sections of the MO were triple-labeled with anti–h-αS, anti-RFP, and anti-SOD2. Colocalization of h-αS and hM3D was seen in DMnX neurons from control mice, whereas all three proteins were coexpressed within DMnX neurons of SOD2 animals; representative images of two cells from a control mouse (top two rows) and two neurons from an SOD2 animal (bottom rows). Scale bars, 10 μm. (**F**) Control and SOD2 animals were treated with daily injections of saline or CNO during weeks 4 and 5 after AAV injection. They were then sacrificed at the end of week 5. Medullary tissue sections were labeled with anti–c-fos; representative images show an area of the dorsal MO where the central canal and DMnX are delineated with dashed lines. Scale bars, 100 μm. Box and whisker plots show median, upper and lower quartiles, and maximum and minimum as whiskers.

The next set of experiments was aimed at directly testing the role of oxidant stress as a mechanism underlying hyperactivity-induced h-αS spreading. More specifically, these experiments were designed to determine whether enhanced expression of the superoxide scavenging enzyme SOD2 would be effective in counteracting ROS/RNS accumulation and thus preventing h-αS transfer from hyperactive neurons. To achieve the expression of h-αS, activity-inducing DREADD and SOD2, a group of iR26-αS mice (SOD2 group) received a single injection of a solution containing three different viral vectors into the left vagus nerve; Cre- and hM3D^fl^-AAVs were injected together with AAVs carrying human SOD2 DNA under the control of the CAG promoter (Fig. 8A). A separate group of animals (control group) also received an intravagal injection of a cocktail of three viral vectors consisting of Cre- and hM3D^fl^-AAVs together with empty AAVs lacking protein coding sequence (Fig. 8A). Mice in the control and SOD2 groups were further divided into two experimental groups and treated with either saline or CNO. Daily saline/CNO administrations were carried out during weeks 4 and 5 after vagal AAV injection; animals were then sacrificed at the end of week 5.

Initial analyses were carried out to assess transgene expression and to verify CNO-induced hyperactivity. Levels of exogenous h-αS, endogenous mouse αS, and total αS (human plus mouse) mRNAs were analyzed by quantitative real-time PCR (qPCR) and compared in the dorsal MO of control versus SOD2 mice. Data showed comparable αS (exogenous, endogenous, or total) expression between the two experimental groups (Fig. 8B). Immunohistochemistry was carried out to assess expression of h-αS and hM3D proteins in medullary sections that were stained with either anti–h-αS or anti-RFP. Microscopic examination and densitometric analysis of the stained sections showed comparable h-αS or RFP immunoreactivity in samples from control and SOD2 mice; either of the two proteins was specifically expressed in the DMnX-MO, consistent with targeted Cre and hM3D transduction after AAV vagal administration (Fig. 8, C and D). Coexpression was further evaluated in medullary tissue sections triple-stained with anti–h-αS, anti-RFP, and anti-SOD2. Coimmunoreactivity for h-αS and hM3D, but not SOD2, was observed in samples from control mice, whereas coexpression of h-αS, hM3D, and SOD2 was evident within DMnX neurons from SOD2 animals (Fig. 8E). The effectiveness of hM3D stimulation and consequent induction of hyperactivity was assayed in medullary tissue sections stained with anti–c-fos. Comparative analyses were done in samples from control and SOD2 mice treated with saline or CNO. Data showed a marked increase in DMnX c-fos expression in all samples from CNO-treated animals, indicating robust and comparable hM3D stimulation and neuronal hyperactivity regardless of whether vagal injections with Cre- and hM3D^fl^-AAVs were associated with coadministration of empty or SOD2-carrying viral vectors (Fig. 8F).

**Fig. 9.**
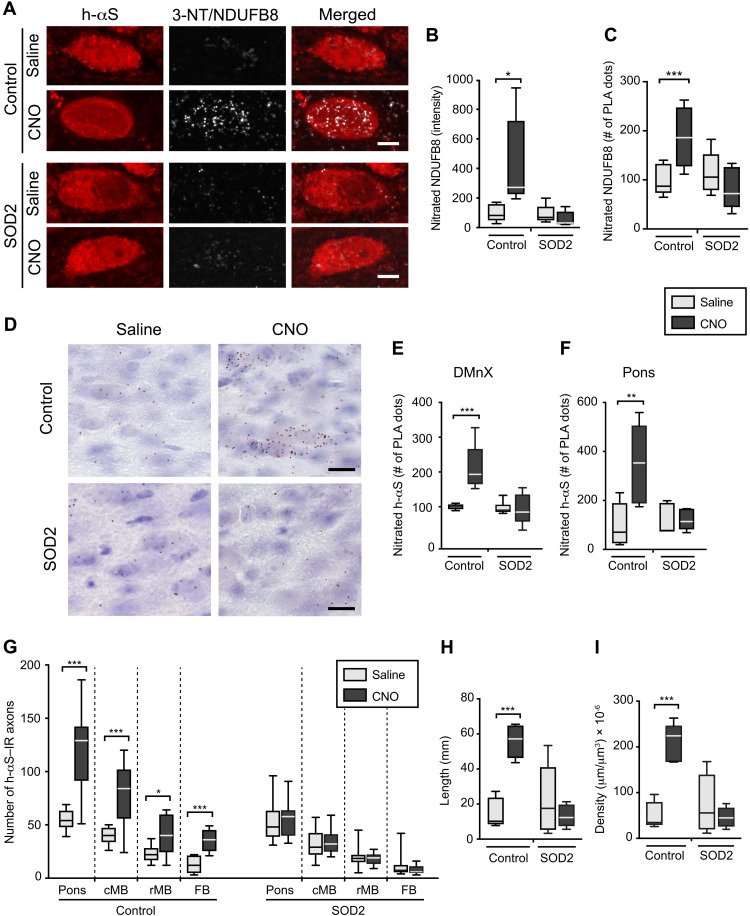
SOD2 overexpression prevents protein nitration and h-αS spreading during neuronal hyperactivity. Mice were coinjected with Cre-, hM3D^fl^-, and empty-AAVs (control mice) or Cre-, hM3D^fl^-, and SOD2-AAVs (SOD2 animals). Animals were also treated with saline (gray) or CNO (black). (**A** to **C**) Mice were divided into four groups: control/saline (*n* = 5), control/CNO (*n* = 5), SOD2/saline (*n* = 5), and SOD2/CNO (*n* = 5). Sections of the MO were processed for 3-NT/NDUFB8 PLA and also labeled with anti–h-αS. Images show DMnX neurons. Scale bars, 10 μm (A). Fluorescent intensity and number of PLA dots were measured within h-αS–positive DMnX neurons; 10 to 15 neurons per animal were analyzed, and for each animal, intensity values and PLA counts were averaged. Data are expressed as percentage of the mean value in the corresponding saline-injected group (B and C). (**D** to **F**) Four groups (*n* ≥ 5 per group) of mice were treated as above. To detect nitrated h-αS, sections of the MO and pons were processed for 3-NT/h-αS PLA and counterstained with Nissl. PLA dots are shown in the left DMnX. Scale bars, 50 μm (D). For each animal, the number of PLA dots was counted in the left DMnX (E) and in left pontine sections (F); data were calculated as percentage of the mean value in the corresponding saline-injected group. (**G**) Four groups (*n* ≥ 7 per group) of mice were treated as above. The number of h-αS–immunoreactive axons was counted in sections of the left pons, caudal midbrain, rostral midbrain, and forebrain immunostained with anti–h-αS. (**H** and **I)** The length (H) and density (I) of h-αS–immunoreactive axons were measured in the pons (*n* = 5 per group). Plots show median, upper and lower quartiles, and maximum and minimum as whiskers. *P < 0.05, **P < 0.01, and ***P < 0.001.

Formation and accumulation of nitrated proteins were compared during hyperactivity in the absence or presence of human SOD2 expression. Medullary tissue sections were processed for 3-NT/NDUFB8 or 3-NT/h-αS PLA and analyzed using fluorescent or bright-field detection. In the DMnX of control mice, PLA signals for nitrated NDUFB8 and nitrated h-αS were noticeably affected by hM3D stimulation; microscopic observation (Fig. 9, A and D), measurement of fluorescent intensity (Fig. 9B), and count of PLA dots (Fig. 9, C and E) all revealed CNO-induced increases in neuronal labeling. These findings sharply contrasted to the results in SOD2 animals in which levels of NDUFB8 and h-αS nitration remained unchanged after CNO administration (Fig. 9, A to E). In a parallel set of analyses, h-αS nitration was also compared in tissue sections of the pons from control and SOD2 mice with or without CNO treatment. Samples were processed for 3-NT/h-αS PLA with bright-field detection, and the number of chromogenic PLA dots was counted using unbiased stereology. PLA counts were found to be significantly increased in pontine sections from control animals injected with CNO. They were instead quite similar in samples from the SOD2 treatment group after administration of either saline or CNO (Fig. 9F).

Caudo-rostral h-αS spreading was finally evaluated in the pons, midbrain, and forebrain of saline- and CNO-injected control and SOD2 mice. Coronal brain sections were stained with anti–h-αS, and the number, length, and density of h-αS–immunoreactive axons were compared under these different experimental conditions. Axonal counts were significantly higher in all brain sections from control animals injected with CNO, whereas CNO administration had no effect on the number of h-αS–positive axons in samples from SOD2 mice (Fig. 9G). Moreover, measurements of length and density of h-αS–loaded axons in pontine sections yielded values that were significantly increased after hM3D stimulation in control but not SOD2 animals (Fig. 9, H and I). Together, these data provided direct evidence of a protective effect of ROS scavenging against h-αS spreading and thus indicated a key role of ROS and RNS accumulation in promoting the transfer of h-αS from hyperactive neurons.

### Hyperactivity promoted h-αS aggregation, and this effect was reversed by SOD2 expression

**Fig. 10.**
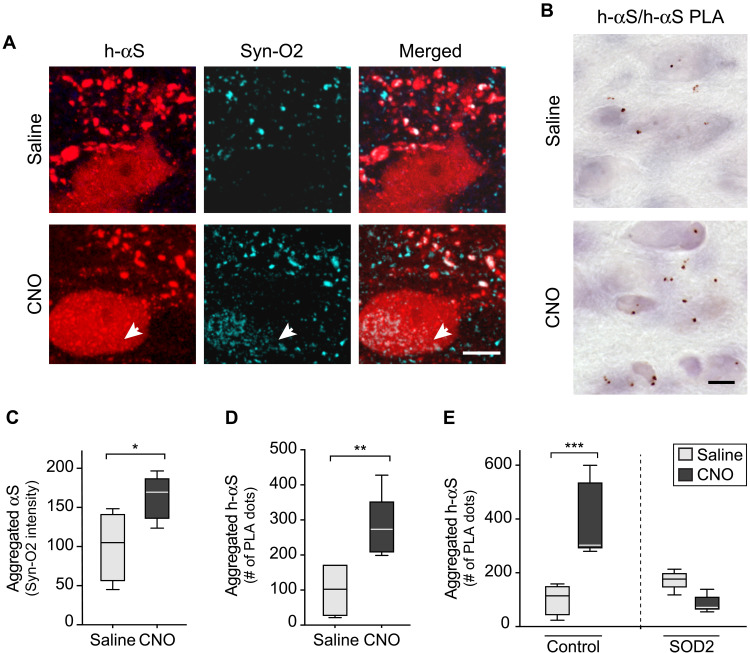
SOD2 overexpression prevents hyperactivity-induced h-αS aggregation. (**A** and **B**) Mice received an injection of a solution containing Cre- and hM3D^fl^-AAVs into the left vagus nerve. They were then divided into two groups and treated with saline or CNO. Medullary sections from mice treated with saline (*n* = 4) or CNO (*n* = 5) were double-stained with anti–h-αS and Syn-O2, an antibody recognizing aggregated αS forms. Images show neurites and cell bodies in the left DMnX; the arrow indicates a cell body colabeled for h-αS and Syn-O2. Scale bar, 10 μm (A). Sections of the MO from mice treated with saline (*n* = 5) or CNO (*n* = 5) were processed for h-αS/h-αS PLA. Images show PLA dots in the left DMnX. Scale bar, 20 μm (B). (**C**) Analysis of sections stained with anti–h-αS and Syn-O2. Syn-O2 fluorescent intensity was measured within h-αS–positive DMnX neurons; an average of 30 neurons per animal was analyzed. Cell intensity values were averaged for each animal, and data were calculated as percentage of the mean value in the saline-injected group. (**D**) Analysis of sections processed for h-αS/h-αS PLA. For each mouse, the number of PLA dots was counted in the left DMnX; data were calculated as percentage of the mean value in the corresponding saline group (D). (**E**) Mice were coinjected with Cre-, hM3D^fl^-, and empty-AAVs (control mice) or Cre-, hM3D^fl^-, and SOD2-AAVs (SOD2 animals). Animals were also treated with saline or CNO. Sections of the MO from control/saline (*n* = 5), control/CNO (*n* = 5), SOD2/saline (*n* = 5), and SOD2/CNO (*n* = 5) mice were processed for h-αS/h-αS PLA. The number of PLA dots was counted in the left DMnX; data were calculated as percentage of the mean value in the corresponding saline-injected group. Plots show median, upper and lower quartiles, and maximum and minimum as whiskers. *P < 0.05, **P < 0.01, and ***P < 0.001.

Enhanced neuronal activity has been reported to promote αS aggregation (*21*). Earlier studies also indicate that the tendency of αS to aggregate may be exacerbated by oxidant stress (*27*, *47*–*49*). Whether a relationship exists between neuronal activity, oxidant stress, and protein aggregation remains elusive, however. Here, a series of experiments were designed to address this question and to assess h-αS aggregation in the absence and presence of neuronal stimulation as well as in the absence and presence of SOD2 expression. First, iR26-αS mice were injected with Cre-/hM3D^fl^-AAVs, treated with saline or CNO for 2 weeks (weeks 4 and 5 after AAV treatment), and sacrificed at the end of week 5. Protein aggregation was assayed using a conformation-specific antibody, Syn-O2, capable of detecting aggregated but not monomeric αS (*27*, *40*, *50*). Further analyses were carried out using a PLA protocol (h-αS/h-αS PLA) well characterized in human and mouse brain specimens; this protocol allows the detection of aggregated and, in particular, oligomeric forms of the protein with a high degree of sensitivity and specificity (*27*, *40*, *51*). Results were similar in medullary tissue sections stained with Syn-O2 or processed for h-αS/h-αS PLA since, with both assays, enhanced protein aggregation was detected in samples from CNO-treated as compared to saline-treated mice (Fig. 10, A and B). When sections from saline-injected animals were double-stained for h-αS and Syn-O2 fluorescence, Syn-O2 immunoreactivity predominantly labeled neuritic projections and terminals (Fig. 10A). Quite in contrast, Syn-O2 staining was obviously present not only within neurites but also within DMnX cell bodies in sections from CNO-treated mice; quantification of signal intensity within h-αS–expressing neurons yielded significantly higher values after hM3D stimulation (Fig. 10, A and C). Signal quantification was also carried out in the DMnX of PLA-processed samples; increased counts of h-αS/h-αS PLA dots in sections from CNO-injected mice provided additional evidence of enhanced aggregation after neuronal stimulation (Fig. 10D).

To investigate the involvement of oxidant stress in hyperactivity-induced h-αS assembly, iR26-αS mice received an intravagal cocktail injection of either Cre-, hM3D^fl^-, and empty-AAVs (control mice) or Cre-, hM3D^fl^-, and SOD2-AAVs (SOD2 mice). Both control and SOD2 mice were also treated with saline or CNO as described above. Medullary tissue sections from all these animals were processed for h-αS/h-αS PLA, and the number of chromogenic PLA dots was stereologically counted in the DMnX. Data revealed that dot counts were markedly increased after CNO administration in control mice; they remain unchanged, however, after hM3D stimulation in sections from SOD2 animals (Fig. 10E). Thus, oxidant stress during neuronal hyperactivity, as documented in this study, played an important role in promoting h-αS aggregation that could be counteracted by boosting intraneuronal ROS scavenging.

## DISCUSSION

The vagus nerve can be used as a conduit for delivering viral vectors to the brain (*27*, *31*, *39*, *40*, *52*). In this study, Cre/*loxP* conditional h-αS knock-in mice received an intravagal injection of Cre-AAVs to achieve stable expression of h-αS driven by the endogenous Rosa26 promoter. AAV-induced transduction targeted vagus-associated neurons and remained strictly confined to areas of the MO that are occupied by efferent (the DMnX) and afferent (the NTS) vagal neurons. This anatomical restriction was confirmed by detection of Cre recombinase mRNA in the DMnX-MO but not in other higher brain regions and is in line with the results of earlier investigations in rats and mice injected with viral vectors into the vagus nerve (*39*, *40*). Expression of h-αS protein was also initially restricted to the dorsal MO. Starting a few weeks later, however, neuron-to-neuron protein transfer was indicated by the detection of h-αS in brain regions outside the MO. Overexpressing cells in the DMnX and/or overexpressing afferent projections in the NTS could conceivably act as donor neurons and sources of the spreading protein. However, it is noteworthy that, while a contribution of NTS nerve terminals, albeit possible, warrants further investigation, several lines of evidence are consistent with an important role played by DMnX neurons in h-αS transfer. Earlier studies demonstrated, for example, that caudo-rostral protein spreading triggered by overexpression of h-αS in the dorsal MO was strictly contingent upon the integrity and viability of DMnX neurons; partial or complete loss of these cells resulted in reduction or cessation, respectively, of h-αS advancement (*52*, *53*).

The initial step of the spreading process involved the passage of h-αS between overexpressing donor neurons and recipient axons innervating the dorsal MO and connecting it to higher brain regions. Following this transfer, retrograde protein spreading through axonal projections resulted in a progressive accumulation of h-αS first in pontine, then in midbrain, and finally in forebrain sites. Brain regions with strong, direct connections to the dorsal MO, such as the locus coeruleus, dorsal raphae, hypothalamus, and amygdala, were primarily affected by this secondary (i.e., posttransfer) h-αS burden, supporting a transfer mechanism that involves anatomically interconnected neurons (*54*, *55*). A feature of h-αS pathology in areas affected by the spreading process was the presence of an increasing number of dystrophic axons loaded with the exogenous protein. This observation of axonal burden, which contrasted with the apparent lack of h-αS buildup within neuronal cell bodies, supports the notion that neuronal projections represent earlier and more vulnerable targets of αS accumulation and pathology (*56*–*58*). Crowding and aggregation of h-αS within nerve fibers may promote axonal pathology but, at the same time, limit protein flow and reduce access of h-αS into neuronal perikarya. Furthermore, as discussed below, a dying-back process triggered by axonal injury during h-αS spreading may lead to cell degeneration before and in the absence of overt h-αS accumulation within neuronal cell bodies (*52*).

To induce h-αS expression and, at the same time, stimulate or inhibit neuronal activity, Cre-AAVs were co-administered with either hM3D^fl^- or hM4D^fl^-AAVs into the vagus nerve of iR26-αS mice. DREADD-induced hyper- or hypoactivity had a significant effect on h-αS spreading; upward protein advancement was markedly enhanced after expression and stimulation of hM3D, an excitatory DREADD, whereas expression and stimulation of the inhibitory receptor hM4D were associated with reduced accumulation of h-αS in brain regions rostral to the MO. Together, these findings provide compelling evidence that neuronal activity affects neuron-to-neuron transfer of h-αS and, by doing so, is able to promote or attenuate protein spreading throughout the brain. It is noteworthy that these findings were obtained under pathophysiological conditions (enhanced αS expression) and mimicked pathological features (progressive caudo-rostral diffusion of αS lesions and αS-induced axonal injury) of likely relevance to PD (*2*, *3*, *56*, *59*–*61*). Under these conditions, neuronal activity could play an important pathogenetic role; it could, for example, modulate the extent and severity of αS burden, affect the exchange of toxic αS species, and/or render specific neuronal populations more or less susceptible to protein exchange and accumulation. Protein spreading, once triggered by enhanced h-αS expression within medullary neurons, has been shown to cause neurodegeneration and a pronounced tissue inflammatory response in higher brain regions affected by spreading-induced h-αS accumulation; these effects were observed at 3, 6, and 12 months after the initial protein transfer event (*52*). Thus, neuronal h-αS burden associated with caudo-rostral protein spreading bears significant and long-lasting pathophysiological consequences that could be exacerbated or lessened by hyper- or hypoactivity, respectively.

A key finding of this study was the demonstration of a relationship between neuronal activity and oxidant stress, which led us to interrogate the role of oxidant stress in hyperactivity-induced h-αS spreading. A higher rate of superoxide formation was detected in the form of ox-DHE accumulation within hyperactive DMnX neurons. Further evidence of hyperactivity-induced oxidative reactions was then obtained from analyses of intraneuronal proteins carrying nitrative modifications. In particular, we evaluated and quantified activity-dependent changes in nitrated h-αS, NDUFB8, and SOD2. During oxidant stress, reaction of superoxide with nitric oxide can generate highly unstable peroxynitrite anions that can in turn react with tyrosine residues of proteins (*46*). Oxidative/nitrative reactions have long been known to modify αS in the human brain. In PD and other human synucleinopathies, Lewy inclusions are robustly labeled with antibodies that react against nitrated αS or recognize 3-NT–modified protein residues, underscoring the relevance that any condition capable of affecting αS nitration may have from the pathophysiological standpoint (*62*, *63*). We report here that enhanced production of 3-NT–modified h-αS occurred after neuronal expression and stimulation of hM3D in vivo. On the other hand, hypoactivity that was induced by expression of hM4D and treatment with CNO lowered the intraneuronal burden of nitrated h-αS. Together, these findings indicate a direct relationship between neuronal activity, oxidant stress, and protein nitration and point to neuronal activity as a potential modulator of pathogenetic processes involving nitrated h-αS accumulation.

Mitochondria could conceivably play an important role as sources of increased ROS production during neuronal hyperactivity due to higher energy demand, stimulation of OXPHOS, enhanced leakage of electrons from the ETC, and consequent reduction of molecular oxygen to superoxide (*24*, *25*). Our data support this possibility and provide intriguing evidence of ROS- and RNS-induced mitochondrial damage during neuronal hyperactivity. Being close to sites of superoxide generation, mitochondrial proteins are highly susceptible to oxidative modifications (*64*). It is also noteworthy that a mitochondrial form of nitric oxide synthase (mitoNOS) could significantly contribute to mitochondrial protein nitration. This mitoNOS is stimulated by calcium influx into mitochondria, and its activity has been proposed to generate larger amounts of NO in neurons with calcium-dependent autonomous pacemaking, including DMnX neurons (*30*, *65*). In our study, increased production of ROS and RNS and pronounced nitration of mitochondrial proteins were indicated by an accumulation of nitrated NDUFB8 and SOD2 and represented an important feature of neuronal hyperactivity. The NDUFB8 subunit is central to complex I assembly, stability, and function that could be severely affected by nitrative reactions (*66*, *67*). Similarly, peroxynitrite-dependent nitration of SOD2 leads to enzyme inactivation (*68*, *69*). Thus, accumulation of these nitrated proteins during neuronal hyperactivity could ultimately set off a self-amplifying toxic loop: superoxide and nitric oxide react together to generate the nitrated proteins, and this nitration may in turn exacerbate superoxide leakage from the ETC (via complex I inhibition) while impairing mitochondrial ROS scavenging (via SOD2 inactivation). Hyperactivity-induced mitochondrial protein nitration (in particular, NDUFB8 nitration) was observed not only in brain tissue from iR26-αS mice coinjected with Cre- and hM3D^fl^-AAVs but also in samples from animals treated with non–Cre-dependent hM3D-AAVs. These findings indicate that hyperactivity itself is capable of promoting mitochondrial nitrative reactions and that overexpression of h-αS is not a necessary requirement for the protein-modifying effect of neuronal stimulation to become overt. It is noteworthy, however, that, although hyperactivity-induced mitochondrial protein nitration may occur in the absence or presence of h-αS accumulation, the toxic consequences of these nitrative modifications are likely to be more severe within h-αS–loaded neurons. This worsening effect may arise, for example, from direct interactions between αS and mitochondria that could exacerbate mitochondrial dysfunction within hyperactive neurons and, together with oxidative/nitrative reactions, ultimately contribute to neuronal injury (*70*, *71*).

Increased superoxide production and enhanced protein nitration indicated the occurrence of oxidant stress within hyperactive neurons and raised interesting pathophysiological scenarios. These findings did not specifically demonstrate, however, that oxidant stress was a primary mechanism responsible for hyperactivity-induced h-αS spreading. Rescue experiments were therefore designed to determine whether this interneuronal protein transfer could be counteracted by preventing ROS/RNS accumulation. We also aimed at further evaluating the relationship between hyperactivity, ROS production, and mitochondrial damage, and for this reason, the strategy for these experiments was to enhance neuronal expression of the mitochondrial superoxide scavenging enzyme SOD2. SOD2 transduction was achieved together with h-αS and hM3D expression via vagal coadministration of Cre-, hM3D^fl^-, and SOD2-AAVs. Transgene tissue expression and cellular protein colocalization were carefully verified to confirm efficacy and reliability of this triple-treatment paradigm. Proper assessment of the effects of human SOD2 expression was also ensured by comparing data in the group of animals injected with Cre-, hM3D^fl^-, and SOD2-AAVs (SOD2 mice) versus another group of mice that received Cre- and hM3D^fl^-AAVs together with empty vectors (control mice). Stimulation of hM3D receptors by CNO treatment caused neuronal hyperactivity in both control and SOD2 mice. The consequences of this hyperactivity were markedly different, however, between these two groups of animals. In control mice, hM3D stimulation was associated with oxidant stress and enhanced h-αS spreading. Quite in contrast, levels of nitrated h-αS and nitrated NDUFB8 remained unchanged despite CNO-induced hyperactivity in SOD2 animals; similarly, hyperactivity had no significant effect on h-αS transfer in the presence of human SOD2 expression. Thus, ROS scavenging by SOD2 protected against the pro-oxidant and nitrative effects of neuronal stimulation and, at the same time, was sufficient to completely prevent hyperactivity-induced h-αS spreading. An important additional finding obtained from these experiments concerns the relationship between neuronal hyperactivity, oxidant stress, and protein aggregation. While stimulation of hM3D receptors in control mice resulted in enhanced h-αS assembly, the same treatment had no effect on protein aggregation in neurons with increased SOD2 expression. Data therefore indicate that oxidant stress not only exacerbates activity-dependent protein spreading but also mediates, at least in part, the development of aggregate pathology within hyperactive neurons.

Different h-αS species, including posttranslationally modified forms of the protein, are likely to have distinct interneuronal mobility and therefore play a more or less pronounced role in spreading processes. On the basis of these premises, accumulation of 3-NT–modified h-αS, as seen in this study, warrants careful evaluation and bears significant implications. Here, we found that nitrated h-αS, detected by 3-NT/h-αS PLA, was present within donor neurons in the DMnX-MO as well as recipient axons in the pons. During neuronal hyperactivity, enhanced protein spreading was associated with increased levels of 3-NT–modified h-αS in the DMnX-MO and pons. Quite in contrast, both h-αS transfer and levels of nitrated h-αS were significantly lowered as a result of neuronal hypoactivity. Last, when SOD2 expression counteracted hyperactivity-induced oxidant stress, decreased h-αS transfer was paralleled by a reduction of nitrated protein within both medullary and pontine neurons. Together, these findings underscore a strict relationship between neuronal activity, oxidant stress, nitrated h-αS burden, and protein spreading. They also suggest that detection of 3-NT–modified αS not only is a marker of oxidant stress but also may characterize neurons that, under pro-oxidant conditions such as those triggered by hyperactivity, become more active sites of h-αS transfer. Another, not mutually exclusive interpretation of our present results is suggested by data of an earlier investigation, showing that nitrative modifications may generate h-αS species with greater propensity to pass from cell to cell (*27*). Nitrated h-αS, with its high motility, could therefore directly participate in interneuronal protein exchanges, and its enhanced formation during neuronal hyperactivity could itself contribute to protein spreading exacerbation.

In summary, experimental evidence presented here reveals a mechanistic link between neuronal activity, oxidant stress, and αS pathology in the form of protein spreading and aggregation. Our in vivo data also identify mitochondria as key targets of oxidant stress and likely sources of ROS/RNS production within hyperactive neurons. The feasibility of protective intervention against hyperactivity-induced αS pathology is demonstrated by the results of rescue experiments; these results show that protein spreading and aggregation can be effectively counteracted by enhancing neuronal ROS scavenging capabilities. Last, evidence from this study underscores the significance of nitrated αS accumulation as a marker of neurons with greater susceptibility to αS transfer and supports a direct involvement of nitrated αS in the spreading process. Further work is warranted to develop and evaluate protective strategies aimed at counteracting nitrated αS burden; this targeted intervention may mitigate αS pathology during neuronal hyperactivity and under other pathophysiological conditions associated with oxidative/nitrative neuronal injury.

## MATERIALS AND METHODS

### Generation of iR26-αS mice

Wild-type human h-αS cDNA (pCR2.1-Topo-αS) was cloned into a Rosa26 targeting vector (PolyGene) downstream to a loxP-flanked neo/STOP cassette. The SacI-linearized vector was electroporated into C57Bl/6N-derived embryonic stem cells, and G418-resistant clones were isolated and screened for correct homologous recombination. Selected clones were injected into gray C57Bl/6 blastocysts, and blastocysts were transferred into CD-1 foster mice for the generation of chimeric animals. Chimeric mice were then bred to C57Bl/6N mice to pass the mutation through the germ line and to obtain homozygous transgenics on a C57Bl/6N background. The following mixture of three primers was used for genotyping purposes: 5′-GCTGTGCTCCACGTTGTCAC-3′, 5′-GGAAAGCTGGGCTTGCATCTC-3′, and 5′-GGAGCGGCGATACCGTAAAG-3′.

The reaction resulted in two bands: one band of 512 base pairs (bp), which indicated integration of the neomycin cassette, and a second band (control fragment) of 380 bp. Homozygous transgenics were viable and fertile. Maintenance and expansion of the colony were achieved through breeding of homozygous mating pairs. Homozygous animals were used for all experiments.

### Viral vectors

All AAVs used in this study were generated using a backbone plasmid of AAV2-derived genome encapsulated into an AAV6 capsid. Most AAVs contained a woodchuck hepatitis virus posttranscriptional regulatory element (WPRE) and a polyadenylation signal sequence downstream to the promoter and transgene sequences. Cre-AAVs lacked the WPRE sequence, and empty-AAV had no transgene protein coding sequence. Production and titration of the AAVs were carried out by Vector Biolabs (hM3D-, hM3D^fl^-, hM4D^fl^-, SOD2-, and empty-AAVs) or Sirion Biotech (Cre-AAVs). High-titer stock AAV preparations were diluted with phosphate-buffered saline. Final titers were (i) 1 × 10^12^ gc/ml, 2 × 10^12^ gc/ml, or 4 × 10^12^ gc/ml for Cre-AAVs; (ii) 3 × 10^12^ gc/ml for hM3D-, hM3D^fl^-, and hM4D^fl^-AAVs; and (iii) 2 × 10^12^ gc/ml for SOD2- and empty-AAVs.

### Animal procedures and tissue processing

Animal experiments were approved by the State Agency for Nature, Environment and Consumer Protection in North Rhine Westphalia, Germany. Experiments were conducted in female and male iR26-αS mice between 15 and 22 weeks of age. Animals were housed in individually ventilated cages, in a specific pathogen–free facility, and kept on a 12-hour light/dark cycle with ad libitum access to food and water. To induce expression of transgene(s) in the DMnX, a solution containing a single AAV preparation or multiple AAVs was injected into the left vagus nerve. Mice were anaesthetized with isoflurane, a small incision was made at the midline of the neck, and the left vagus nerve was isolated (*40*). The AAV-containing solution (800 nl) was injected at a flow rate of 350 nl/min using a 35-gauge blunt steel needle fitted onto a 10-μl NanoFil syringe. After injection, the needle was kept in place for one additional minute to avoid backflow. To stimulate DREADDs, CNO (Tocris) was administered intraperitoneally at a dose of 1 mg/kg dissolved in 0.9% saline. DHE (Abcam) was dissolved in saline/dimethyl sulfoxide (DMSO) (1:1 ratio) and injected intraperitoneally at a dose of 15 mg/kg. Animals were monitored daily throughout the duration of the experiments. No overt changes in body weight, basic motility, and general welfare were noticed as consequences of the surgical procedures or any of the experimental treatments. At the end of the experiments, mice were sacrificed with an injection of sodium pentobarbital (600 mg/kg) (intraperitoneally) and perfused through the ascending aorta with 4% (w/v) paraformaldehyde. Brains were removed and immersed in 4% paraformaldehyde for 24 hours before cryopreservation in 30% (w/v) sucrose. Subsequent analyses were carried out on coronal sections (35 μm) of the brain that were obtained using a freezing microtome.

### Immunohistochemistry with bright-field detection and density analysis

For bright-field microscopy, free-floating sections were quenched by incubation in a mixture of 3% H_2_O_2_ and 10% methanol in tris-buffered saline (pH 7.6). Nonspecific binding sites were blocked by incubation in 5% normal serum. Samples were kept overnight at room temperature in a solution containing the primary antibody: rabbit anti–h-αS (1:50,000; ab138501, Abcam), rabbit anti-RFP (1:20,000; 600-401-379, Rockland), mouse anti–Cre recombinase (1:4000; MAB3120, Millipore), rabbit anti–c-fos (1:2000; 2250, Cell Signaling Technology), and rabbit anti-SOD2 (1:5000; ADI-SOD-110, Enzo Life Sciences). Sections were rinsed and incubated in biotinylated secondary antibody solution (1:200; Vector Laboratories). Following treatment with avidin-biotin–horseradish peroxidase complex (ABC Elite kit, Vector Laboratories), color reaction was developed using a 3,3′-diaminobenzidine kit with or without nickel (Vector Laboratories). Sections were mounted on coated slides, coverslipped with Depex (Sigma-Aldrich), and imaged using a Zeiss Observer.Z1 Microscope (Carl Zeiss) equipped with a motorized stage and AxioCam MRm camera (Carl Zeiss). For bright-field density measurements, slides containing MO sections were scanned (AxioScan.Z1, Carl Zeiss). The DMnX was delineated on three equally spaced sections, and integrated density values were obtained using Fiji (ImageJ version 2.1.0/1.53c).

### Immunofluorescence

For single fluorescent labeling, samples were blocked with 5% normal serum and incubated overnight at 4°C with rabbit anti–h-αS (1:3000). For double fluorescence, sections were first incubated overnight with either rabbit anti-RFP (1:3000) or mouse anti–Syn-O2 (1:2000; TAB-0748CLV, Creative Biolabs) and then incubated with anti–h-αS. Labeling of these primary antibodies was achieved using a secondary antibody conjugated with DyLight 488 or DyLight 594 (1:300; Vector Laboratories). Sections were rinsed, mounted on coated slides, and coverslipped with Vectamount mounting medium (Vector Laboratories). For triple fluorescence, tissue sections were processed using the following sequential labeling procedures. First, for detection of mCherry-conjugated hM3D, mCherry was labeled by incubations with rabbit anti-RFP (1:3000), donkey anti-rabbit Fab fragment (1:200; Jackson ImmunoResearch), and goat anti-donkey Alexa Fluor 594 (1:300; Abcam). Second, SOD2 was labeled by incubations with rabbit anti-SOD2 (1:1000) and goat anti-rabbit Alexa Fluor 647 (1:300, Abcam). Last, h-αS was labeled by overnight incubation with rabbit anti–h-αS conjugated with Alexa Fluor 488 (1:400; ab216124, Abcam). Fluorescence images were collected on Zeiss microscopes (LSM700, LSM800, LSM880, or LSM900) using the ZEN software (Carl Zeiss).

### Axonal counts and quantification of spreading

The total number of h-αS–immunoreactive axons was counted using sections of the left pons, caudal midbrain, rostral midbrain, and forebrain at predefined bregma levels: −5.40 mm (pons), −4.60 mm (caudal midbrain), −3.40 mm (rostral midbrain), and −2.18 mm (forebrain). For measurements of axonal length and density, three equally spaced pontine sections (bregma −5.68, −5.51, and −5.34 mm) were used. After delineation of an area encompassing the locus coeruleus and parabrachial nucleus, measurements were performed using the Space Balls stereological probe (Stereo Investigator software, version 9, MBF Biosciences) (*40*). High-magnification images of h-αS–immunoreactive axons were analyzed on an Observer.Z1 microscope (Carl Zeiss), using a 63× Plan-Apochromat objective.

### Ox-DHE and Syn-O2 quantification

Fluorescence intensity analyses were performed on DMnX-containing medullary sections using an LSM800 or LSM880 confocal microscope. Confocal z-stack images were collected and analyzed with Imaris 9.5 software. Ox-DHE fluorescent signal was acquired on three sections per animal, as previously described (*27*). Briefly, a three-dimensional (3D) surface rendering model of h-αS–immunoreactive DMnX neurons was created. A second 3D surface was created for ox-DHE puncta by applying a constant intensity threshold and was then filtered through h-αS–positive neuronal surfaces. This allowed for specific detection of ox-DHE puncta within immunoreactive neurons and for quantification of these puncta on a per-cell basis. Syn-O2 fluorescence intensity was quantified on 2D images that were generated from 5-μm-thick z-stack images using maximum intensity projection function of the Zen software (Carl Zeiss). Neuronal cell bodies immunoreactive for h-αS were selected by applying a size exclusion filter (120 μm^2^), and Syn-O2 intensity was quantified within these cells.

### In situ proximity ligase assay

Free-floating medullary sections containing the DMnX were processed using Duolink In-Situ PLA (Sigma-Aldrich) according to the manufacturer’s protocols. Aggregated h-αS was detected using a previously described “direct” PLA method (*27*, *51*). This method involved an overnight incubation of sections in solutions containing PLA probes (1:120) directly conjugated to a h-αS–specific antibody (mouse anti-Syn211, Millipore). Detection of nitrated h-αS, nitrated NDUFB8, and nitrated SOD2 was performed using “indirect” PLA. For this assay, sections were first incubated overnight in a solution containing two primary antibodies and then incubated with secondary antibodies conjugated with oligonucleotide probes. For detection of nitrated h-αS, the two antibodies were mouse anti–3-NT (1:250; ab61392, Abcam) and rabbit anti–h-αS (1:4000) (*27*). For detection of nitrated NDUFB8, the two antibodies were mouse anti–3-NT (1:250) and rabbit anti-NDUFB8 (1:300; 14794, Proteintech), and for detection of nitrated SOD2, they were mouse anti–3-NT (1:250) and rabbit anti-SOD2 (1:1000; ADI-SOD-110, Enzo Life Sciences). Samples were then incubated with secondary antibodies conjugated with oligonucleotide probes, namely, anti-rabbit PLUS and anti-mouse MINUS (Duolink, Sigma-Aldrich). Following ligation and amplification, specific PLA signals were visualized using a bright-field or fluorescence detection kit (Duolink, Sigma-Aldrich). As negative controls, sections were processed using the same procedures with the exception that anti–h-αS, anti-NDUFB8, or anti-SOD2 was omitted. In these control specimens, no PLA signal was present.

Bright-field detection was used for assessment of nitrated (3-NT/h-αS PLA) or aggregated (h-αS/h-αS PLA) h-αS in the left DMnX. In these sections, which were counterstained with hematoxylin, quantification of PLA dots was obtained by stereological counts using the optical dissector (Stereo Investigator, MBF Bioscience). The DMnX was delineated at low magnification (10× objective) on every 10th section between bregma −6.96 and −8.00 mm. Coefficients of error of the PLA counts were less than 0.10. Bright-field detection was also used for visualization and quantification of nitrated h-αS in the left pons. For each animal, the number of PLA dots was counted on a single pontine section at the level of bregma −5.34 mm using the meander scan function of Stereo Investigator (MBF Biosciences). For double fluorescent labeling detecting PLA (nitrated NDUFB8 or nitrated SOD2) and h-αS or RFP, the PLA signal was first detected using the Duolink Green Detection Kit (Sigma-Aldrich). Then, sections were incubated with anti–h-αS or anti-RFP, and labeling was detected using DyLight 594 (1:300). Quantification of the fluorescent PLA signal within h-αS–positive DMnX neurons was performed on a single section at the level of the obex. Quantification of the PLA signal within RFP-immunoreactive neurons was carried out on six equally spaced DMnX-containing MO sections. Confocal z-stack images (4 μm thick) were acquired with a 63× Plan-Apochromat objective using an LSM 700 or LSM 900 scanning confocal microscope (Carl Zeiss). Maximum intensity projection images were generated with the ZEN software and, on these images, DMnX neurons immunoreactive for h-αS were delineated using Fiji (ImageJ version 2.1.0/1.53c). A fixed intensity threshold was set for the delineation of PLA dots that was automatically done using the “analyze particles” function of ImageJ. Intensity of the PLA signal and number of PLA dots were quantified on a per-cell basis.

### RT-PCR and qPCR analyses

Tissue dissection, RNA extraction, and cDNA preparation were carried out as previously described (*40*). For RT-PCR assessment of Cre recombinase expression, mRNA was extracted from tissue specimens of the left (ipsilateral to the AAV injection) dorsal MO, pons, and caudal midbrain. For each sample, cDNA amplification was done using Power SYBR Green Master Mix (Applied Biosystems) and forward and reverse primers specific for Cre (i) and Hprt [housekeeping control, (ii)]: (i) 5′-CGCGGTCTGGCAGTAAAAAC and 5′-CGCCGCATAACCAGTGAAAC and (ii) 5′-TCCTCCTCAGACCGCTTTT and 5′-CCTGGTTCATCATCGCTAATC.

PCR products were mixed with 6× sample buffer (New England Biolabs) with 5% DMSO and loaded on a 2.0% SeaKem agarose gel (Lonza Bioscience) pre-stained with RedSafe dye (1:20,000, Intron Biotechnology). Images were acquired with an InGenius3 imaging system and GeneSys software (Syngene). qPCR measurements of human, mouse, and total αS RNA expression were carried out on tissue extracts from the left dorsal MO. Samples were analyzed with a StepOnePlus Real-Time PCR instrument (Applied Biosystems). Triplicate measurements of cDNA (2.5 ng) were done using Power SYBR Green Master Mix (Applied Biosystems) and forward and reverse primers specific for h-αS (i), mouse αS (ii), total αS (iii), and Hprt (see above): (i) 5′-AATGAAGAAGGAGCCCCACAG and 5′-AAGGCATTTCATAAGCCTCATTGTC, (ii) 5′-AGTGGAGGGAGCTGGGAATATAG and 5′-CCAGGATTCCTTCCTGTGGGTAC, and (iii) 5′-GCTCAGAAGACAGTGGAGGG and 5′-TCTTCCAGAATTCCTTCCTGTGGG. Fold change expression levels were calculated using the 2^−ΔΔCT^ method.

### Statistical analysis

Unless specified differently in the figure legends, at least five animals per treatment group were used for all histological, biochemical, and molecular biology analyses. Statistical analyses were performed with GraphPad Prism (8.0) using unpaired *t* test for axonal counts/measurements and PLA stereological counts, and nonparametric Mann-Whitney *U* test for intensity image analyses and qPCR measurements. Analysis of variance (ANOVA) followed by Tukey post hoc test was used when comparisons were made among four treatment groups. *P* values of less than 0.05 were considered statistically significant.

## Notes

View/request a protocol for this paper from *Bio-protocol*.
