# Supplementary material for: Neuronal hyperactivity–induced oxidant stress promotes in vivo α-synuclein brain spreading
Source: Sci Adv. 2022 Aug 31;8(35):eabn0356. doi: 10.1126/sciadv.abn0356 (PMC9432848; doi:10.1126/sciadv.abn0356)
Supplement: Supplementary file 1 — Figs. S1 and S2 Data S1 [file sciadv.abn0356_sm.pdf]

Science Advances
AAAS

Supplementary Materials for

Neuronal hyperactivity–induced oxidant stress promotes in vivo α-synuclein brain spreading

Michael Helwig *et al.*

Corresponding author: Donato A. Di Monte, donato.dimonte@dzne.de

Sci. Adv. **8**, eabn0356 (2022)
DOI: 10.1126/sciadv.abn0356

The PDF file includes:

Figs. S1 and S2

Data S1

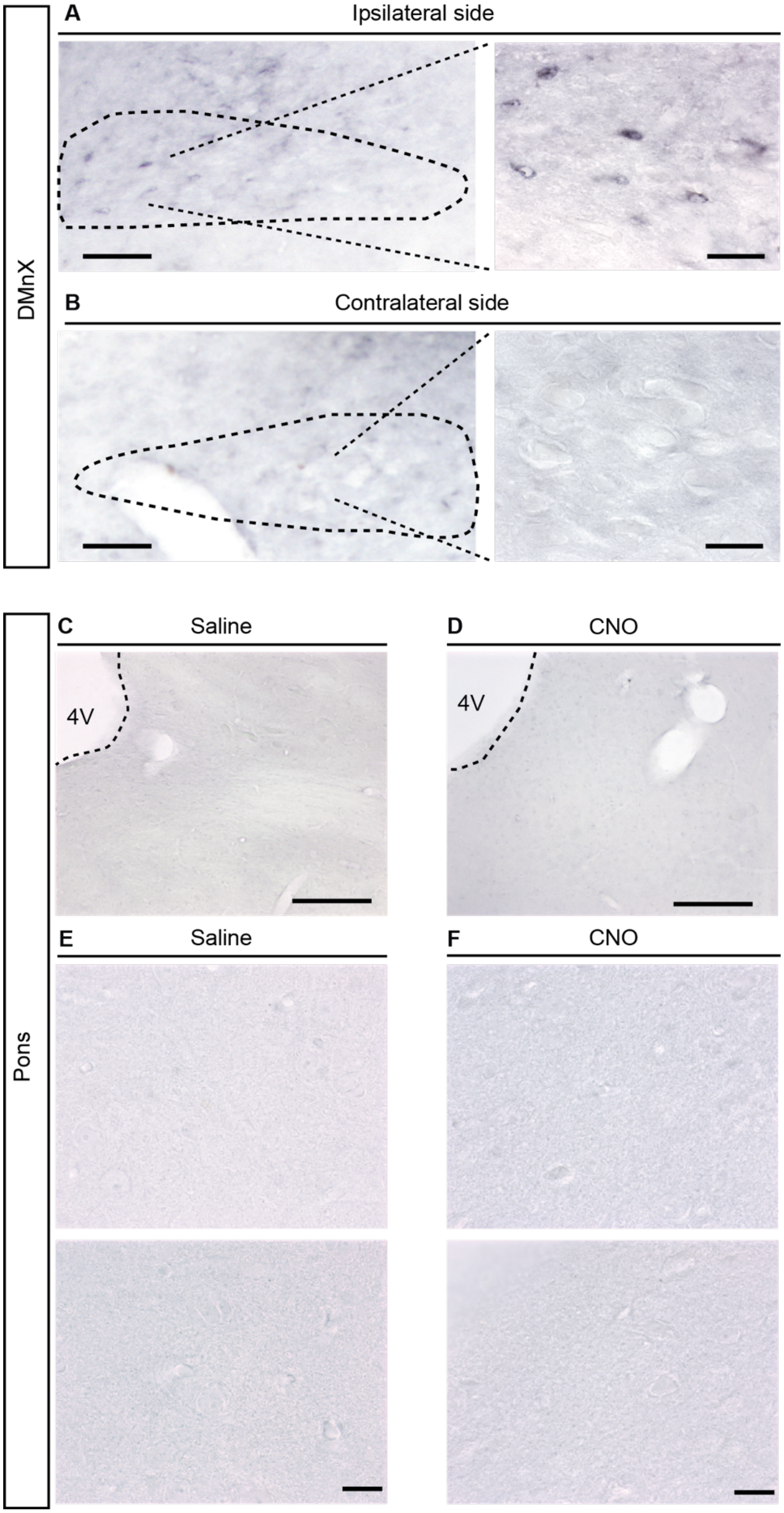
DMnX
A
Ipsilateral side
B
Contralateral side
Pons
C
Saline
D
CNO
4V
4V
E
Saline
F
CNO

Fig. S1. Selective detection of Cre-recombinase protein in the DMnX. (A-F) iR26-aS mice received an intravagal injection of Cre-AAVs together with hM3Dfl-AAVs and were sacrificed five weeks later. They were also treated with daily intraperitoneal injections of either saline or CNO for two weeks prior to sacrifice. Tissue sections from the MO (A and B) and pons (C-F) were immunostained with anti-Cre and processed for brightfield microscopy. (**A** and **B**) Representative sections of the dorsal MO show the presence of immunoreactive neurons in the left (ipsilateral to the vagal AAV injection, A) but not the right (contralateral to the AAV injection, B) DMnX (delineated with dashed lines). Images were collected at low (left panels) and higher (right panels) magnification. Scale bars, 50 and 20 μm in the left and right panels, respectively. (**C**-**F**) Representative sections of the left pons at low (C and D) and higher (E and F) magnification from either saline- (C and E) or CNO- (D and F) treated mice show lack of Cre protein immunoreactivity. The 4th ventricle (4V) is delineated with dashed lines in C and D. Scale bars, 200 μm in C and D, and 20 μm in E and F.

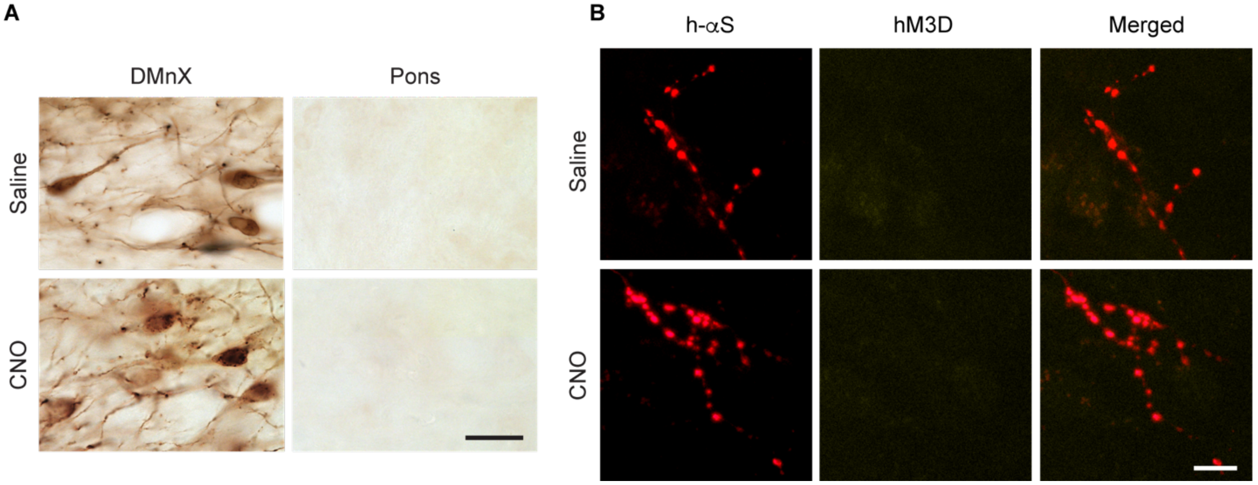

Fig. S2. Selective detection of mCherry-fused hM3D protein in the dorsal MO. (**A** and **B**) iR26-αS mice received an intravagal injection of Cre-AAVs together with hM3D^{fl}-AAVs and were sacrificed five weeks later. They were also treated with daily intraperitoneal injections of either saline or CNO for two weeks prior to sacrifice. (**A**) Tissue sections from the medulla oblongata and pons were immunostained with anti-RFP (for the detection of mCherry-tagged hM3D) and processed for brightfield microscopy. Images of representative sections show that robust protein expression characterized cell bodies and neurites in the DMnX, whereas pontine sections were devoid of immunoreactivity. Scale bar, 40 µm. (**B**) Tissue sections from the pons were double-labeled with anti-h-αS (red) and anti-RFP (yellow) and processed for confocal microscopy. Representative images show axons loaded with h-αS as a result of the spreading process. The same axons were devoid of RFP immunoreactivity, further confirming lack of medullary-to-pons transfer of the RFP protein. Scale bar, 10 µm.

Data S1. All quantitative data used in this manuscript are listed below

Fiber counts

Fig. 2C

Sample	Titer (gc/ml)	Pons	cMB	rMB	FB
1	1x10E12	10	3	0	0
2	1x10E12	2	0	0	0
3	1x10E12	3	1	0	0
4	1x10E12	2	0	0	0
1	2x10E12	10	27	11	1
2	2x10E12	41	29	14	2
3	2x10E12	24	19	10	0
4	2x10E12	32	23	11	2
5	2x10E12	40	28	15	1
1	4x10E12	46	34	14	2
2	4x10E12	59	40	17	3
3	4x10E12	81	57	24	7
4	4x10E12	52	37	16	3
5	4x10E12	66	46	18	5

Fig. 2G

Sample	Time point	Pons	cMB	rMB	FB
1	4 weeks	14	5	1	0
2	4 weeks	33	25	14	3
3	4 weeks	39	27	20	6
4	4 weeks	27	19	12	2
5	4 weeks	23	17	11	3
6	4 weeks	19	7	5	4
1	5 weeks	77	63	38	20
2	5 weeks	37	28	21	8
3	5 weeks	45	32	28	16
4	5 weeks	59	39	24	12
5	5 weeks	48	29	19	10
6	5 weeks	63	44	27	15
1	6 weeks	91	81	50	31
2	6 weeks	44	30	21	17
3	6 weeks	88	73	41	24

4	6 weeks	40	31	24	13
5	6 weeks	105	82	57	35

Fig 3H

Sample	Treatment group	Pons	cMB	rMB	FB
1	Saline	52	44	30	4
2	Saline	75	52	24	7
3	Saline	50	39	19	3
4	Saline	65	50	27	10
5	Saline	29	21	11	10
6	Saline	51	37	16	13
1	CNO	127	99	48	47
2	CNO	221	154	47	69
3	CNO	161	101	59	37
4	CNO	243	163	58	22
5	CNO	192	87	36	22
6	CNO	114	38	17	8
7	CNO	118	63	22	16

Fig. 7D

Sample	Treatment group	Pons	cMB	rMB	FB
1	Saline	64	49	22	11
2	Saline	58	27	27	8
3	Saline	66	45	23	14
4	Saline	73	42	28	14
5	Saline	66	37	19	11
6	Saline	44	32	18	8
7	Saline	57	35	27	12
8	Saline	75	46	29	21
1	CNO	41	22	19	6
2	CNO	32	27	15	3
3	CNO	37	23	17	5
4	CNO	41	28	18	3
5	CNO	30	19	10	5
6	CNO	27	18	11	2
7	CNO	40	27	12	5
8	CNO	47	29	20	14
9	CNO	36	24	18	8

10	CNO	39	23	16	10

Fig. 9G

Sample	Treatment group	Pons	cMB	rMB	FB
1	Control Saline	39	26	12	13
2	Control Saline	69	43	22	22
3	Control Saline	54	40	37	21
4	Control Saline	53	40	19	5
5	Control Saline	48	34	17	3
6	Control Saline	60	47	23	7
7	Control Saline	63	50	28	12
1	Control CNO	96	85	26	28
2	Control CNO	87	63	23	35
3	Control CNO	186	93	40	49
4	Control CNO	144	56	29	23
5	Control CNO	51	24	12	21
6	Control CNO	123	83	61	42
7	Control CNO	140	101	52	40
8	Control CNO	138	120	64	48
9	Control CNO	129	104	58	36
1	SOD2 Saline	46	31	19	12
2	SOD2 Saline	96	55	45	42
3	SOD2 Saline	80	57	16	4
4	SOD2 Saline	31	17	9	6
5	SOD2 Saline	60	12	5	5
6	SOD2 Saline	72	49	25	14
7	SOD2 Saline	48	32	19	6
8	SOD2 Saline	39	27	17	4
9	SOD2 Saline	54	37	21	11
10	SOD2 Saline	39	24	17	8
11	SOD2 Saline	57	40	24	13
12	SOD2 Saline	48	27	18	7
13	SOD2 Saline	33	16	13	6
14	SOD2 Saline	44	25	20	8
1	SOD2 CNO	34	24	9	6
2	SOD2 CNO	61	21	16	3

3	SOD2 CNO	91	59	19	16
4	SOD2 CNO	67	47	27	8
5	SOD2 CNO	63	42	20	9
6	SOD2 CNO	43	28	14	3
7	SOD2 CNO	62	32	19	6
8	SOD2 CNO	58	35	23	12
9	SOD2 CNO	37	26	13	5
10	SOD2 CNO	64	40	23	10
11	SOD2 CNO	33	20	12	5
12	SOD2 CNO	49	26	15	6
13	SOD2 CNO	51	33	21	13

Fiber Length Measurements

Fig. 2D

Sample	Titer	Length
1	$1 X 10^{12}$	3,32247681
2	$1 X 10^{12}$	2,8478374
3	$1 X 10^{12}$	2,03416968
4	$1 X 10^{12}$	1,62733569
1	$2 X 10^{12}$	12,40843
2	$2 X 10^{12}$	19,12119
3	$2 X 10^{12}$	9,01815
4	$2 X 10^{12}$	9,96743
5	$2 X 10^{12}$	14,848
1	$4 X 10^{12}$	29,49546
2	$4 X 10^{12}$	19,12119
3	$4 X 10^{12}$	38,85264
4	$4 X 10^{12}$	18,51094
5	$4 X 10^{12}$	23,59637

Fig. 2H

Sample	Time point	Length
1	4w	9,49279
2	4w	12,34063

3	4w	12,81527
4	4w	6,17031445
5	4w	6,64495361
6	4w	5,96567432
1	5w	17,79898
2	5w	10,91671
3	5w	15,18847
4	5w	17,08702
5	5w	17,087
6	5w	21,3588
1	6w	25,39321
2	6w	21,5961
3	6w	33,93673
4	6w	15,4258
5	6w	29,4277

Fig. 3I

Sample	Treatment group	Length
1	Saline	8,78083
2	Saline	13,76455
3	Saline	51,4984
4	Saline	41,0563
5	Saline	11,3913
6	Saline	16,3751
1	CNO	47,7013
2	CNO	63,12706
3	CNO	36,78457
4	CNO	62,4151
5	CNO	43,4295
6	CNO	25,1559
7	CNO	38,2085

Fig. 9H

Sample	Treatment group	Length
1	Control Saline	19,93486
2	Control Saline	10,20475
3	Control Saline	7,83155
4	Control Saline	9,49279
5	Control Saline	27,29178
1	Control CNO	63,83902
2	Control CNO	49,1252
3	Control CNO	65,50026
4	Control CNO	43,66684
5	Control CNO	57,194
1	SOD2 Saline	17,56166
2	SOD2 Saline	53,3969
3	SOD2 Saline	28,47838
4	SOD2 Saline	7,59423
5	SOD2 Saline	3,32248
1	SOD2 CNO	17,79898
2	SOD2 CNO	12,34063
3	SOD2 CNO	5,69567
4	SOD2 CNO	21,35878
5	SOD2 CNO	9,49279

Fiber Density Measurements

Fig. 2D

Sample	Titer	Density
1	1×10^{12}	9,92625631
2	1×10^{12}	9,00823821
3	1×10^{12}	6,65698967
4	1×10^{12}	5,45183871
1	2×10^{12}	33,1159927
2	2×10^{12}	64,5669858
3	2×10^{12}	29,5410695

4	2 X 10^{12}	27,2808213
5	2 X 10^{12}	50,225453
1	4 X 10^{12}	93,5416515
2	4 X 10^{12}	61,4027668
3	4 X 10^{12}	101,237278
4	4 X 10^{12}	46,6505544
5	4 X 10^{12}	54,5992355

Fig. 2H

Sample	Time point	Density
1	4w	31,7398631
2	4w	43,1948183
3	4w	43,1314852
4	4w	20,3368241
5	4w	23,2587448
6	4w	21,0557175
1	5w	64,2546217
2	5w	39,186562
3	5w	53,7205683
4	5w	60,8746241
5	5w	58,8107743
6	5w	77,5465451
1	6w	88,2766161
2	6w	70,917796
3	6w	110,367298
4	6w	49,2953606
5	6w	104,911586

Fig. 3I

Sample	Treatment group	Density
1	Saline	30,29356
2	Saline	49,62487
3	Saline	181,5401
4	Saline	147,8718

5	Saline	42,43834
6	Saline	57,6237
1	CNO	166,3533
2	CNO	197,8378
3	CNO	116,739
4	CNO	197,3981
5	CNO	155,2162
6	CNO	88,48645
7	CNO	140,2919

Fig. 9I

Sample	Treatment group	Treatment
1	Control Saline	62,01022
2	Control Saline	34,51013
3	Control Saline	26,1299
4	Control Saline	34,15471
5	Control Saline	96,1293
1	Control CNO	224,1468
2	Control CNO	167,892
3	Control CNO	262,993
4	Control CNO	167,254
5	Control CNO	229,3966
1	SOD2 Saline	55,83816
2	SOD2 Saline	168,0115
3	SOD2 Saline	109,6157
4	SOD2 Saline	28,84502
5	SOD2 Saline	11,27526
1	SOD2 CNO	56,88156
2	SOD2 CNO	44,84469
3	SOD2 CNO	19,78838
4	SOD2 CNO	76,04995
5	SOD2 CNO	33,09114

Stereological analyses

Fig. 4D

Sample	Treatment group	Number of dots
1	Saline	177,108992
2	Saline	169,612767
3	Saline	73,9310328
4	Saline	57,7634789
5	Saline	65,5166005
6	Saline	56,0624819
1	CNO	357,277127
2	CNO	417,724325
3	CNO	608,093941
4	CNO	299,67733
5	CNO	243,07584
6	CNO	366,857371
7	CNO	310,503678

Fig. 4E

Sample	Treatment group	Number of dots
1	Saline	62,2578819
2	Saline	117,191282
3	Saline	135,502433
4	Saline	111,697948
5	Saline	93,9971964
6	Saline	79,3482861
1	CNO	236,824111
2	CNO	164,800254
3	CNO	235,603364
4	CNO	261,849344
5	CNO	95,2179433
6	CNO	178,838817
7	CNO	285,65383

Fig. 7C

Sample	Treatment group	Number of dots
1	Saline	158,643626
2	Saline	75,5636038
3	Saline	109,27173

4	Saline	137,342732
5	Saline	67,4083023
6	Saline	51,7663148
1	CNO	39,048134
2	CNO	53,5525662
3	CNO	55,9393281
4	CNO	58,1142039
5	CNO	52,7223027
6	CNO	62,4465715

Fig. 9E

Sample	Treatment group	Number of dots
1	Control Saline	101,679431
2	Control Saline	99,0743688
3	Control Saline	89,0996803
4	Control Saline	110,128123
5	Control Saline	100,001524
1	Control CNO	203,379151
2	Control CNO	178,807946
3	Control CNO	327,408768
4	Control CNO	152,378042
5	Control CNO	193,347052
1	SOD2 Saline	95,2510716
2	SOD2 Saline	80,5941825
3	SOD2 Saline	87,386634
4	SOD2 Saline	89,8578324
5	SOD2 Saline	90,4148221
6	SOD2 Saline	132,345112
1	SOD2 CNO	32,5454378
2	SOD2 CNO	66,8625842
3	SOD2 CNO	100,063097
4	SOD2 CNO	69,5996053
5	SOD2 CNO	127,759641
6	SOD2 CNO	154,851165

Fig. 9F

Sample	Treatment group	Number of dots
1	Control Saline	144,658236
2	Control Saline	70,3581021

3	Control Saline	34,1383169
4	Control Saline	19,4717995
5	Control Saline	231,348926
1	Control CNO	353,872202
2	Control CNO	204,022158
3	Control CNO	559,159803
4	Control CNO	451,208594
5	Control CNO	174,854521
1	SOD2 Saline	74,1568935
2	SOD2 Saline	198,970457
3	SOD2 Saline	74,1568935
4	SOD2 Saline	77,4355416
5	SOD2 Saline	179,017681
1	SOD2 CNO	163,458594
2	SOD2 CNO	68,6929275
3	SOD2 CNO	166,591987
4	SOD2 CNO	98,1797225
5	SOD2 CNO	114,368845

Fig. 10D

Sample	Treatment group	Number of dots
1	Saline	102,371429
2	Saline	31,8105263
3	Saline	172,353885
4	Saline	21,3994987
5	Saline	172,030075
1	CNO	273,568421
2	CNO	277,617043
3	CNO	427,992982
4	CNO	198,814536
5	CNO	216,309774

Fig. 10E

Sample	Treatment group	Number of dots
1	Control Saline	158,807517
2	Control Saline	60,944642
3	Control Saline	24,0264093
4	Control Saline	141,813611
5	Control Saline	114,271204

1	Control CNO	600,070086
2	Control CNO	471,930422
3	Control CNO	302,118842
4	Control CNO	280,241239
5	Control CNO	303,160488
1	SOD2 Saline	213,566785
2	SOD2 Saline	184,396648
3	SOD2 Saline	117,722194
4	SOD2 Saline	177,104114
5	SOD2 Saline	173,978669
1	SOD2 CNO	138,558151
2	SOD2 CNO	55,2148299
3	SOD2 CNO	70,8415439
4	SOD2 CNO	83,3433215
5	SOD2 CNO	71,8831894

Brightfield staining density measurements

Fig. 3F

Sample	Treatment group	integrated density
1	Saline	87,04653
2	Saline	115,2659
3	Saline	97,68753
4	Saline	139,9699
5	Saline	97,55126
6	Saline	101,4426
7	Saline	109,4317
8	Saline	51,60461
1	CNO	265,6267
2	CNO	233,2854
3	CNO	218,0514
4	CNO	294,8751
5	CNO	133,4128
6	CNO	159,9978
7	CNO	154,8667
8	CNO	278,9642

Fig. 8C

Sample	Treatment group	integrated density
1	Control	103,228072
2	Control	98,9160815
3	Control	91,3175991
4	Control	117,054479
5	Control	89,4837573
6	Control	89,1138157
7	Control	105,279905
8	Control	99,0177411
9	Control	102,31727
10	Control	95,9042037
1	SOD2	104,265236
2	SOD2	117,878433
3	SOD2	103,152025
4	SOD2	105,279905
5	SOD2	104,343983
6	SOD2	109,511216
7	SOD2	102,363426
8	SOD2	115,553792
9	SOD2	82,1200038
10	SOD2	92,5926079

Fig. 8D

Sample	Treatment group	integrated density
1	Control	88,9260299
2	Control	101,218006
3	Control	98,8399137
4	Control	116,502561
5	Control	129,319466
6	Control	95,4460325
7	Control	115,239133
8	Control	87,1916222
9	Control	89,4146749
10	Control	92,9897269
11	Control	84,9128343
1	SOD2	93,6235921
2	SOD2	105,692997

3	SOD2	110,671263
4	SOD2	88,5047946
5	SOD2	93,1581006
6	SOD2	103,096811
7	SOD2	110,507521
8	SOD2	81,0403989

Fluorescent intensity measurements and fluorescent PLA dot counts

Fig. 4B

Sample	Treatment group	Intensity
1	Saline	129,13253
2	Saline	123,99547
3	Saline	80,6987952
4	Saline	102,494217
5	Saline	63,6056867
6	Saline	94,1359879
7	Saline	102,041255
8	Saline	103,823477
1	CNO	228,878602
2	CNO	278,102554
3	CNO	269,255711
4	CNO	384,720048
5	CNO	134,893853
6	CNO	101,581819
7	CNO	123,308874

Fig. 5B

Sample	Treatment group	Intensity
1	Saline	74,067086
2	Saline	57,7987421
3	Saline	149,119497
4	Saline	16,0377358
5	Saline	202,93501
1	CNO	1035,01048
2	CNO	2618,44864
3	CNO	1925,36688

4	CNO	581,132075
5	CNO	727,044025

Fig. 5B

Sample	Treatment group	Number of dots
1	Saline	134,433962
2	Saline	77,186964
3	Saline	114,255765
4	Saline	34,067086
5	Saline	139,937107
1	CNO	365,566038
2	CNO	1283,85744
3	CNO	592,243187
4	CNO	315,674891
5	CNO	275,943396

Fig. 5D

Sample	Treatment group	Intensity
1	Saline	155,7350888
2	Saline	211,8442051
3	Saline	55,78495911
4	Saline	43,20766008
5	Saline	33,42309994
1	CNO	381,4083383
2	CNO	241,8112906
3	CNO	1173,583682
4	CNO	443,5816876
5	CNO	302,5234391

Fig. 5D

Sample	Treatment group	Number of dots
1	Saline	167,5
2	Saline	119,15
3	Saline	72,2
4	Saline	82,75
5	Saline	55,9
1	CNO	259,6
2	CNO	242,35

3	CNO	391,1
4	CNO	162,5
5	CNO	130,45

Fig. 6C

Sample	Treatment group	Intensity
1	Saline	81,7274648
2	Saline	66,0387324
3	Saline	111,778169
4	Saline	101,198592
5	Saline	138,264789
1	CNO	102,117254
2	CNO	161,800704
3	CNO	160,262676
4	CNO	159,340845
5	CNO	242,756338

Fig. 9B

Sample	Treatment group	Intensity
1	Control Saline	171,387496
2	Control Saline	81,7740716
3	Control Saline	75,2463853
4	Control Saline	26,5809012
5	Control Saline	145,014663
1	Control CNO	258,468797
2	Control CNO	945,74203
3	Control CNO	497,646619
4	Control CNO	271,844141
5	Control CNO	195,531469
1	SOD2 Saline	62,8872663
2	SOD2 Saline	82,0986029
3	SOD2 Saline	38,7405619
4	SOD2 Saline	68,9561846
5	SOD2 Saline	199,674063
1	SOD2 CNO	70,6550362
2	SOD2 CNO	142,486622
3	SOD2 CNO	31,426377
4	SOD2 CNO	28,6442104

5	SOD2 CNO	20,6106905

Fig. 9C

Sample	Treatment group	Intensity
1	Control Saline	140,379185
2	Control Saline	83,0307919
3	Control Saline	87,4008338
4	Control Saline	64,5421541
5	Control Saline	124,714267
1	Control CNO	143,875219
2	Control CNO	232,889606
3	Control CNO	186,179291
4	Control CNO	111,604142
5	Control CNO	262,5128
1	SOD2 Saline	121,419927
2	SOD2 Saline	105,811897
3	SOD2 Saline	68,5760387
4	SOD2 Saline	90,3590157
5	SOD2 Saline	182,965482
1	SOD2 CNO	133,738791
2	SOD2 CNO	119,154746
3	SOD2 CNO	72,2065349
4	SOD2 CNO	58,3568645
5	SOD2 CNO	31,4643001

Fig. 10C

Sample	Treatment	Intensity
1	Saline	121,517885
2	Saline	88,4460215
3	Saline	148,354608
4	Saline	45,0273978
1	CNO	147,455072
2	CNO	169,39871
3	CNO	196,789355
4	CNO	177,61772
5	CNO	123,704839

qPCR analyses

2-ΔΔCT hα-syn / Hprt Fig. 8B

Sample	Treatment group	2-ΔΔCT
1	Control	0,59
2	Control	1,26
3	Control	1,13
4	Control	0,75
5	Control	0,71
6	Control	1,28
7	Control	1,87
8	Control	0,92
1	SOD2	1,42
2	SOD2	1,3
3	SOD2	0,8
4	SOD2	0,82
5	SOD2	0,73
6	SOD2	2,26

2-ΔΔCT mα-syn / Hprt Fig. 8B

Sample	Treatment group	2-ΔΔCT
1	Control	0,81
2	Control	0,9
3	Control	1,14
4	Control	1,2
5	Control	0,81
6	Control	0,92
7	Control	1,07
8	Control	1,26
1	SOD2	1,17
2	SOD2	1,11
3	SOD2	1,09
4	SOD2	1,17
5	SOD2	1,05
6	SOD2	1,14

2-ΔΔCT hα-syn / Hprt Fig. 8B

Sample	Treatment group	2-ΔΔCT
1	Control	0,71

2	Control	0,95
3	Control	1,1
4	Control	1,22
5	Control	0,73
6	Control	1,08
7	Control	1,15
8	Control	1,21
1	SOD2	1,2
2	SOD2	1,17
3	SOD2	0,91
4	SOD2	1,34
5	SOD2	1,02
6	SOD2	1,44
